# Merging Supramolecular and Covalent Helical Polymers: Four Helices Within a Single Scaffold

**DOI:** 10.1021/jacs.1c10327

**Published:** 2021-12-03

**Authors:** Zulema Fernández, Berta Fernández, Emilio Quiñoá, Félix Freire

**Affiliations:** † Centro Singular de Investigación en Química Biolóxica e Materiais Moleculares (CiQUS) and Departamento de Química Orgánica, Universidade de Santiago de Compostela, 15782 Santiago de Compostela, Spain; ‡ Departamento de Química Física, University of Santiago de Compostela, 15782 Santiago de Compostela, Spain

## Abstract

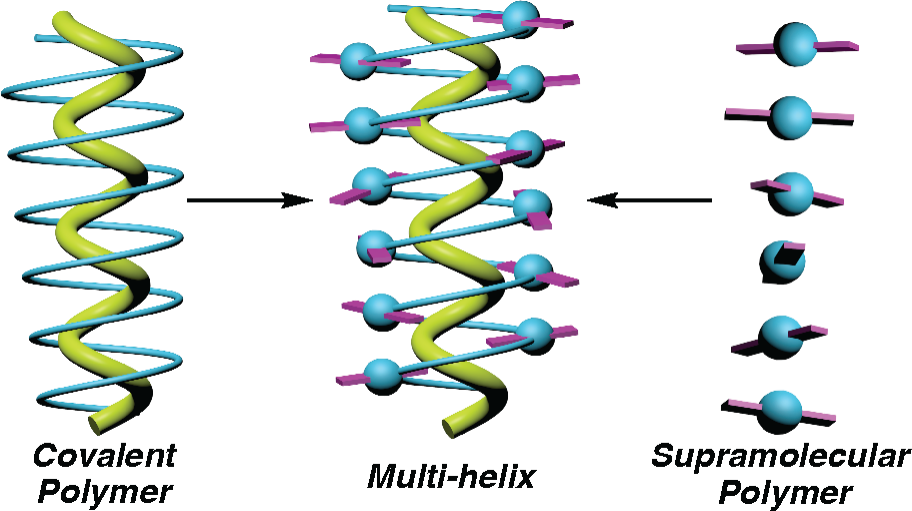

Supramolecular and covalent polymers share multiple structural effects such as chiral amplification, helical inversion, sergeants and soldiers, or majority rules, among others. These features are related to the axial helical structure found in both types of materials, which are responsible for their properties. Herein a novel material combining information and characteristics from both fields of helical polymers, supramolecular (oligo(*p*-phenyleneethynylene) (OPE)) and covalent (poly(acetylene) (PA)), is presented. To achieve this goal, the poly(acetylene) must adopt a dihedral angle between conjugated double bonds (ω1) higher than 165°. In such cases, the tilting degree (Θ) between the OPE units used as pendant groups is close to 11°, like that observed in supramolecular helical arrays of these molecules. Polymerization of oligo[(*p*-phenyleneethynylene)*_n_*]phenylacetylene monomers (*n* = 1, 2) bearing *L*-decyl alaninate as the pendant group yielded the desired scaffolds. These polymers adopt a stretched and almost planar polyene helix, where the OPE units are arranged describing a helical structure. As a result, a novel multihelix material was prepared, the ECD spectra of which are dominated by the OPE axial array.

## Introduction

**Figure 1:**
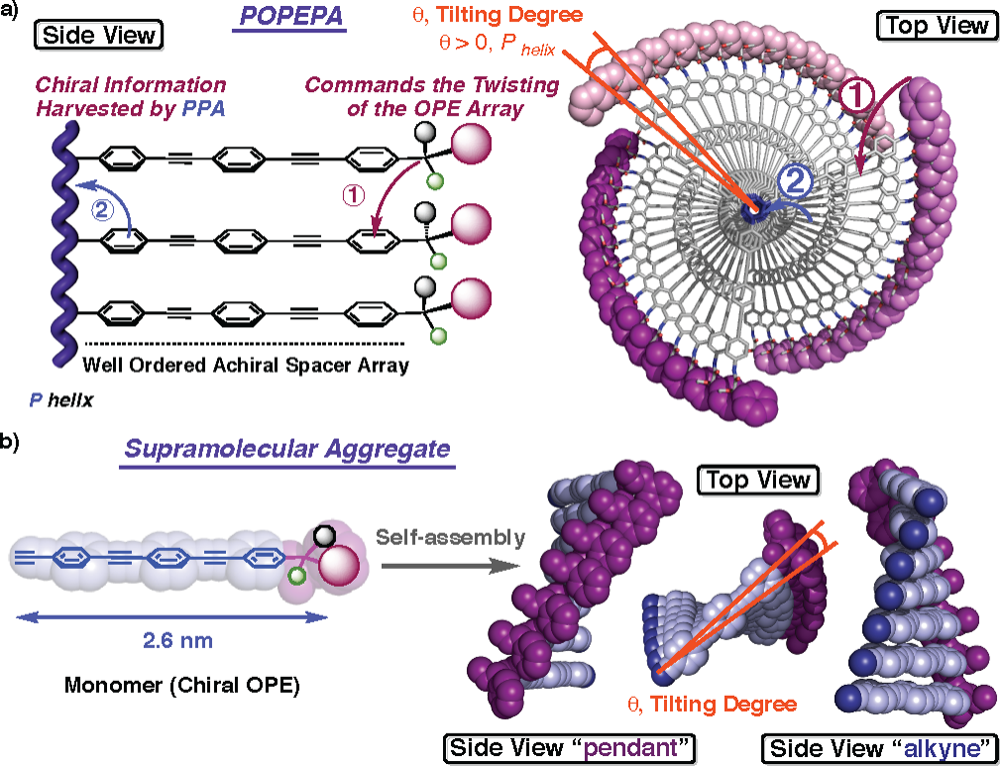
Chiral OPE arrays in (a) covalent and (b) supramolecular helical polymers.

Helices are abundant structural motifs present in nature in many macromolecules such as peptides, proteins, DNA, and polysaccharides and are directly related to the biological functions of these biomolecules.^1−4^ This structure–function relationship led the scientific community to look for novel materials that adopt helical structures, such as covalent and supramolecular helical polymers.^5−20^ Nowadays, it is possible to understand how these polymers are folded and which of the structural features of the building blocks induce covalent or supramolecular polymers to adopt a helical structure. Moreover, these studies led to the development of dynamic helical polymers—covalent and supramolecular—whose helical sense (plus (*P*) or minus (*M*)),^21−26^ elongation (compressed or stretched),^27−31^ or aggregate shape (J-aggregate, H-aggregate, etc.)^32−36^ can be altered by the presence of different external stimuli (e.g., solvent, pH, temperature, metal ions, chiral additives, or light). This dynamics revealed different communication mechanisms between components in both covalent and supramolecular copolymers. Therefore, different chiral amplification or chiral enhancement effects such as sergeants and soldiers, majority rules, chiral coalition, chiral accord, and chiral conflict have been observed.^37−50^ As a consequence, the common properties presented by these polymers have allowed the development of materials combining these two structural motifs.^51−65^ Regarding the helical sense induction mechanism, some research groups have demonstrated the efficient long-distance transmission of chiral information for poly(isocyanide)s,^66,67^ poly(vinylterphenylene)s,^68,69^ and poly(acetylene)s.^70−73^ Recently, our group has reported on poly[oligo(*p*-phenyleneethynylene)acetylene]s (POPEPAs), a novel family of helical polymers.^74^ In these polymers, even though the chiral center is placed at a remote position from the backbone, a helix induction occurs due to the chiral arrangement of the achiral spacers, which is harvested by the polyene backbone. Hence, in a first step the stereogenic centers of the monomer repeating units (mru) command the achiral rigid oligo(*p*-phenyleneethynylene) (OPE) spacers to arrange with a specific tilting degree (Θ), the value of which depends on the absolute configuration of the chiral center. This chiral arrangement of the OPE units, which are stabilized through π–π interactions between them, is then harvested by the polyene backbone, adopting a specific *P* or *M* helical sense (Figure 1a). This chiral arrangement of OPE units in POPEPAs drove us to study the supramolecular self-assembly of the monomer units. Remarkably, it was demonstrated that these monomers arrange into long supramolecular helical polymers, which are stabilized by hydrogen bond interactions between the amide groups of the chiral moieties and π–π interactions among the OPE units (Figure 1b).^75^ The presence of a chiral OPE arrangement in both covalent and supramolecular helical polymer systems attracted our attention, and we thought about the possibility of combining both families of helical polymers within a single helical structure to create a multihelix material.

**Figure 2:**
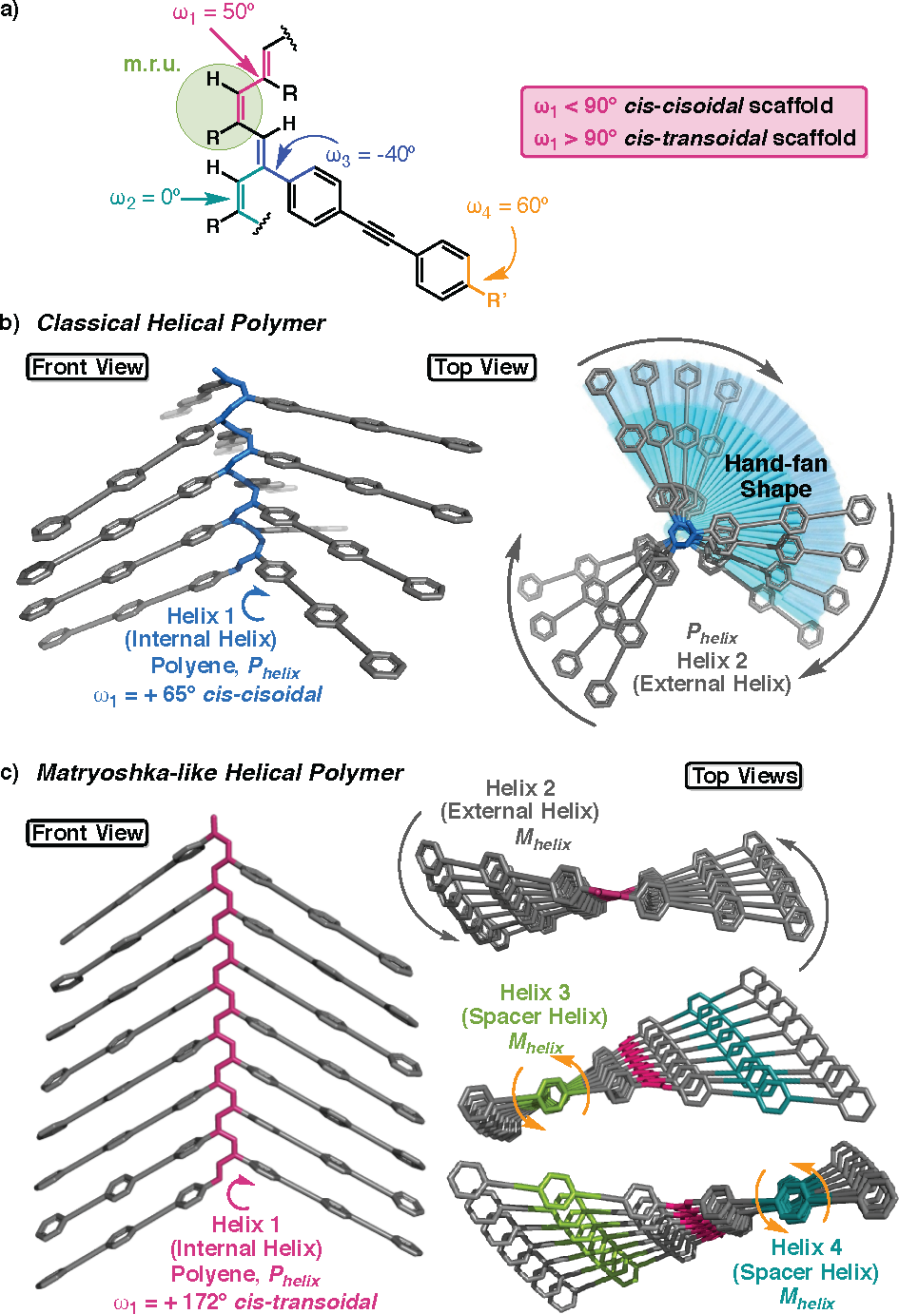
(a) Main dihedral angles involved in the helical structures of PPAs and their derivatives. (b) POPEPA adopting a classical helical structure. (c) POPEPA showing a multihelix scaffold.

To produce these multihelix materials based on POPEPAs, modeling studies varying the main dihedral angles (ω_1_, ω_2_, ω_3_, and ω_4_) (Figure 2a) were first carried out. To this end, different helical structures were built that comprised the requirements of both materials, namely the poly(acetylene) (PA) helix and the supramolecular OPE helix. From these studies it was extracted that it is necessary to control the dihedral angle between conjugated double bonds (ω_1_), which is directly related to the tilting degree adopted by the OPE units, to generate the desired POPEPA. Hence, if POPEPA adopts a *cis–cisoidal* polyene scaffold (ω_1_ < 90°),^76−78^ the classical helix is formed and constituted in turn by two coaxial helices (Figure 2b). In such helical scaffolds, the internal helix is determined by the polyene backbone (helix 1), while the external rotation sense is defined by the helical array of the pendant groups (helix 2) (Figure 2b).^79,80^

The tilting degree (Θ) between OPE units within these helical structures is high, describing a hand-fan-shaped array. As a result, no evidence of a multihelix material should be found in *cis–cisoidal* POPEPAs. Nevertheless, it was found that if POPEPA adopts an extended *cis–transoidal* helical structure with a ω_1_ dihedral angle higher than 160°, the two classical internal and external helices found in PAs (helix 1 and helix 2, respectively) coexist with two other helical scaffolds. These novel helices (helix 3 and 4) correspond to the helical array of the achiral OPE units used as spacers between the chiral pendant and the polyene backbone, and the rotation sense of the helices is coincident with that observed for the outer helix (helix 2) (Figure 2c). It should be pointed out that the helical structures described by the OPE units within *cis*–*transoidal* helical polymers are coincident with those found in an OPE supramolecular helix (Figure 1b). Therefore, using this approach we decided to seek the stabilization of a supramolecular helix within a covalent helical polymer.

## Results and Discussion

**Figure 3:**
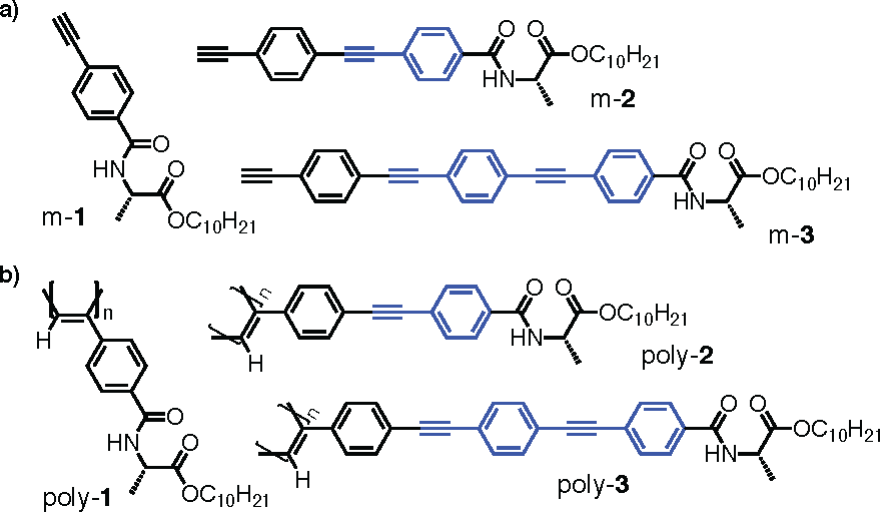
Chemical structures of (a) monomers m-[**1**–**3**] and (b) polymers poly-**1**–**3**.

To perform these studies, it is necessary to design and prepare a POPEPA that adopts a *cis–transoidal* helical scaffold. From the literature, it is known that poly(phenylacetylene)s (PPAs) bearing benzamide connectors between the backbone and the pendant groups promote the formation of *cis–transoidal* structures.^81^ Thus, as a model compound we used the PPA that had the benzamide of *L*-decyl alaninate (poly-**1**, Figure 3b), which adopts a stretched *cis*–*transoidal* (2/1) helix.^82,83^ OPE monomers (*n* = 1, 2) containing the benzamide of the *L*-decyl alaninate, namely decyl (4-((4-ethynylphenyl)ethynyl)benzoyl)-*L*-alaninate and decyl (4-((4-((4-ethynylphenyl)ethynyl)phenyl)ethynyl)benzoyl)-*L*-alaninate (m-[**2**] and m-[**3**], respectively) (Figure 3a), were prepared and further polymerized with a Rh(I) catalyst, which generated poly-[**2**] and poly-[**3**] in good yields and low polydispersity and with a high content of *cis*-double bonds (Figure 3b and Tables S1 and S2). Nevertheless, in these polymers the *cis*-content of the double bonds cannot be quantified with precision due to the large differences in the *T*_2_ NMR values for the different protons of the polymers (see section 3.2 of the Supporting Information).^84^ Next, structural and dynamic behavior studies were carried out for poly-**2** and poly-**3**.

**Figure 4:**
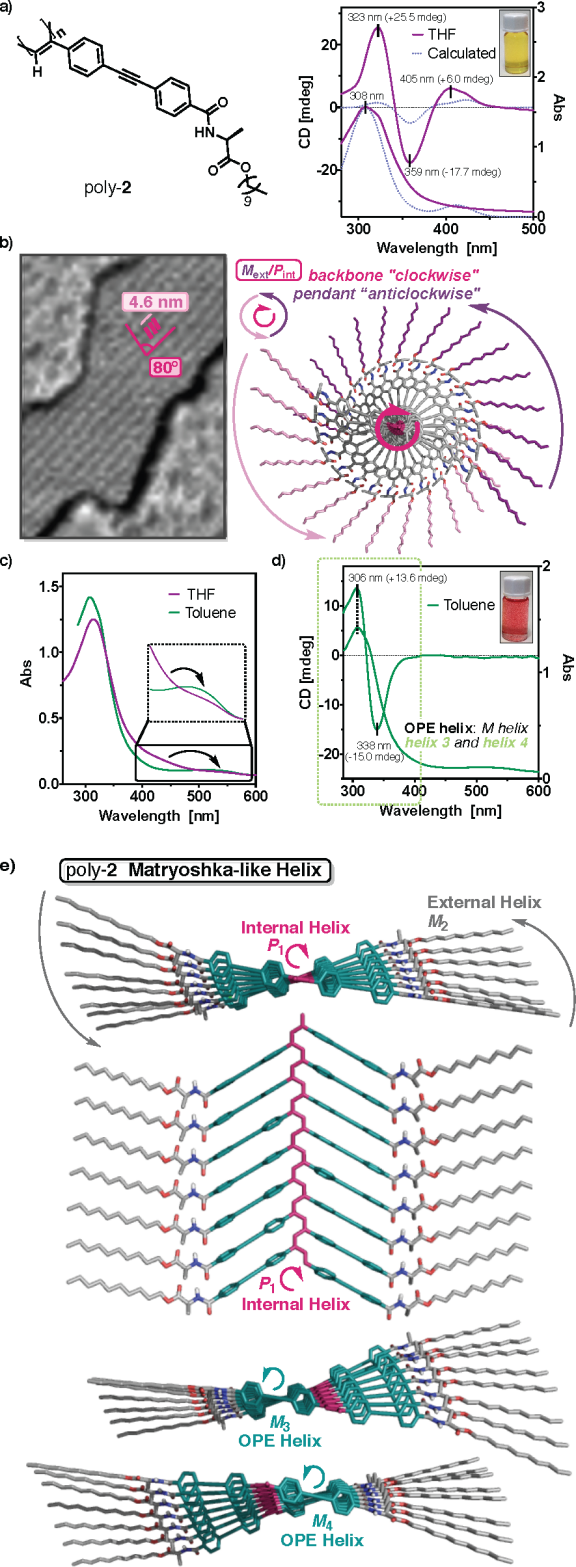
(a) Chemical structure of poly-**2** and ECD and UV–vis spectra (THF, [poly-**2**] = 0.44 mM, 25 °C) compared to the simulated ECD (fwhm = 20 nm) and UV–vis traces. (b) High-resolution AFM images and 3D model of poly-**2** in polar solvents. (c) Comparison of the UV–vis spectra obtained for poly-**2** in CHCl_3_ and toluene. (d) ECD and UV–vis spectra of poly-**2** in toluene ([poly-**2**] = 0.44 mM, 25 °C). (e) 3D model of poly-**2** displaying a multihelix material.

CD studies of poly-**2** in different solvents such as CHCl_3_, DCM, THF, and DMF ([poly-**2**] = 0.44 mM) showed the classical ECD trace of a helical polymer with three alternating Cotton effects (Figures 4a and S21). The first positive Cotton band, which corresponds to the polyene, indicates the presence of a *P* internal helix (Figure 4a). To obtain information related to the orientation of the external helix, AFM studies were performed. A 2D crystal of poly-**2** was prepared from a CHCl_3_ solution by the Langmuir–Schaefer technique^85^ and employing highly oriented pyrolytic graphite (HOPG) as the substrate. The AFM analysis revealed the presence of well-ordered monolayers. From these high-resolution images it was possible to extract the orientation of the external helix, namely the *M* helix, and different structural parameters, such as the helical pitch (4.6 nm) and the packing angle (80°) (Figures 4b and S27).

To corroborate the information obtained from the experimental data, computational studies (TD-DFT(CAM-B3LYP)/3-21)] were performed on the *M* helix (*n* = 8) of poly-**2**, namely the *cis*–*transoidal* skeleton (ω_1_ = 165° and ω_3_ = 80°). In this model, the chiral moiety was introduced in an *antiperiplanar* conformation by placing both carbonyl groups in opposite orientations and, to reduce computational demands, the long alkyl chain was replaced by a methyl group. The simulated ECD spectrum (Figure 4a and the SI for detailed information) is in good agreement with the one obtained experimentally, indicating that the proposed model is a good approximation to the structure described for poly-**2**. Interestingly, when poly-**2** is dissolved in low-polar solvents such as CCl_4_ or toluene, a yellow to red color change occurs that is indicative of helical stretching. UV–vis studies confirmed the elongation of the polyene chain due to a 100 nm bathochromic shift of the vinylic band from 425 (CHCl_3_) to 525 nm (toluene) (Figure 4c). Moreover, the solubility of the polymer decreased in these solvents due to the presence of a highly stretched, almost planar, helix. ECD studies of poly-**2** in CCl_4_ and toluene revealed the disappearance of the classical ECD trace with three alternating Cotton effects, now depicting a large bisignated (∓) signal centered at 323 nm (Figures 4d and S21). Intriguingly, the ECD trace obtained for poly-**2** in CCl_4_ or toluene is coincident with the CD signature of an OPE supramolecular helix, where the ∓ sign of the CD trace is indicative of a *M* helical array of the OPE units within the POPEPA scaffold.^75^ From this information, a molecular model was built for poly-**2** dissolved in low-polarity solvents using a large ω_1_ value (ca., 175°), which corresponded to an almost planar structure (Figure 4e). By looking at this 3D model, it is possible to visualize the two coaxial helices of a PPA, one described by the polyene backbone (*P*_1_, internal helix, ω_1_= +175**°**) and the other described by the pendant groups (*M*_2_, external helix). As expected, the presence of a *cis–transoid* polyene skeleton causes the inner and outer helices to rotate in opposite directions (*P*_1_/*M*_2_). In addition to these two coaxial helices, two other helices can be observed in this 3D model structure, namely *M*_3_ and *M*_4_. They are described by the OPE units used as spacers to separate the chiral pendant group from the polyene backbone (Θ = 11**°**) and show helical senses that coincide with that described by the outer helix *M*_2_. Interestingly, although this structure is made up of four helices going inside out, namely *P*_1_, *M*_3_, *M*_4_, and *M*_2_, the ECD spectrum is governed by the helical array of the OPE units, and the chiroptical information on the polyene chain becomes negligible (ECD null at ca. 525 nm but UV–vis active).

In this helical material where four different helices coexist within the same structure, it is possible to observe how the supramolecular helices described by the pendant groups (helices 2, 3, and 4) are covalently attached to a polyene backbone (the covalent helix (helix 1)) placed in the core of this multihelix material.

**Figure 5:**
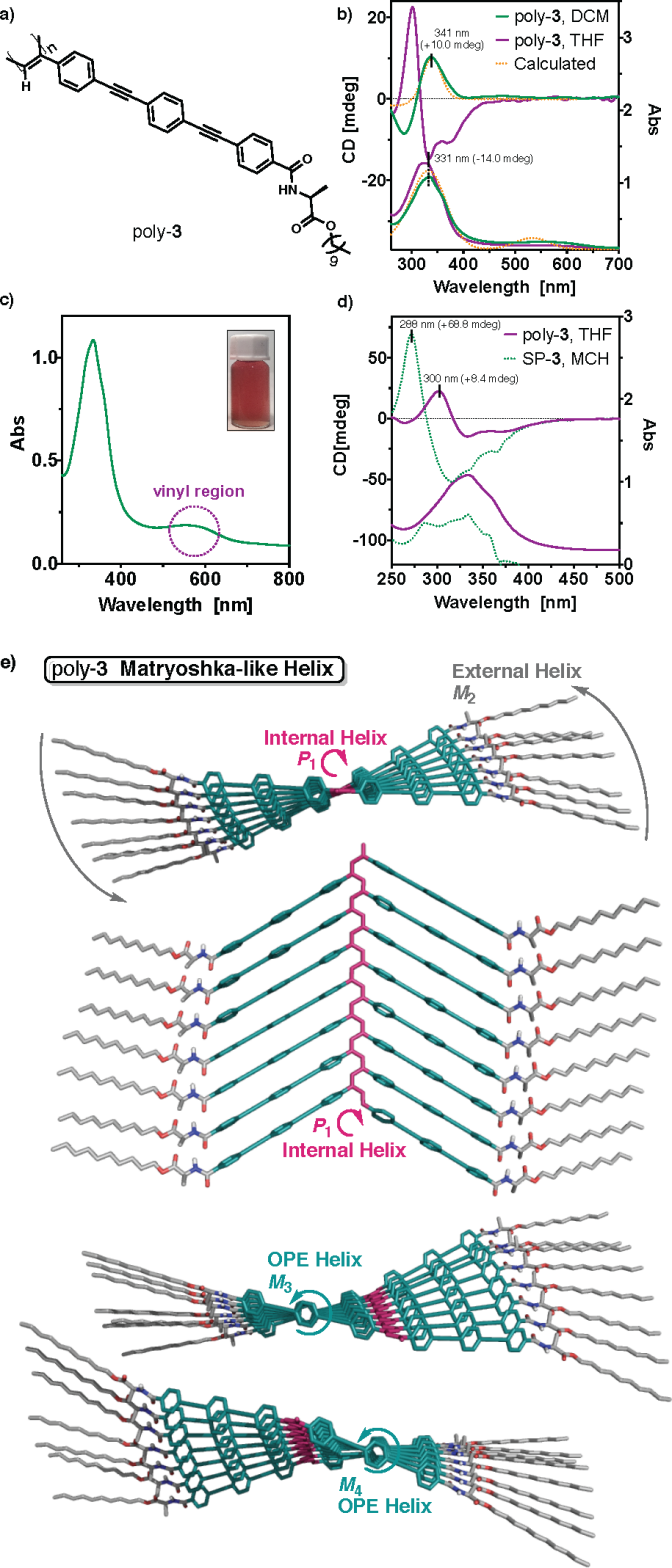
(a) Chemical structure of poly-**3**. (b) Comparison for the experimental (DCM and THF, [poly-**3**] = 0.72 mM, 25 °C) and calculated ECD and UV–vis spectra for poly-**3**; fwhm = 20 nm. (c) UV–vis spectrum of poly-**3** in DCM ([poly-**3**] = 0.72 mM, 25 °C). (d) Comparison of the ECD and UV–vis spectra obtained for poly-**3** (THF, [poly-**3**] = 0.72 mM, 25 °C) and SP-**3** (MCH, [poly-**3**] = 0.15 mM, 25 °C). (e) 3D model of poly-**3** describing a multihelix material.

To demonstrate the versatility and robustness of our hypothesis and results, similar studies were performed for poly-**3**, which has an extra OPE unit in the spacer (*n* = 2).To this end, solutions of poly-**3** ([poly-**3**] = 0.72 mM) were prepared in different solvents such as CCl_4_, CHCl_3_, THF, toluene, ODCB, DCM, and 1,2-DCE (Figures 5a and S24). All these poly-**3** solutions showed deep red colors and poor solubilities, which were indicative of a highly stretched helix with a large hydrophobic surface and a high aggregation tendency.^29^ UV–vis studies of these poly-**3** solutions corroborated the stretched helix due to the presence of the polyene band at ca. 565 nm (Figure 5b and c). In addition, ECD studies for poly-**3** do not show a Cotton band in the polyene region in any of the tested solvents (Figures 5b, d and S24) but instead a strong bisignated signal at shorter wavelengths (ca., 315 nm). The ECD trace obtained is similar to that recorded for the supramolecular helical arrangement of m-[**3**] in low polararity solvents such as MCH (SP-**3**, Figure 5d and section 7 in the Supporting Information). In this case, the bathochromic shift of the OPE band, which is visible in the CD spectrum when poly-**3** is compared with SP-**3**, is attributed to its conjugation with the polyene backbone. These data indicate that poly-**3** constitutes a multihelix material where two novel helices appear due to the chiral arrangement of the OPE units. Hence, four helical structures coexist in this polymer: the internal helix described by the polyene backbone (helix 1), the external helix described by the pendant groups (helix 2), and the helical structures described by the OPE (*n* = 2) units employed as spacers (helix 3 and helix 4). These helices are interconnected and it is possible to extract the orientation of the others by knowing the orientation of one of them (Figure 5e). Therefore, if poly-**3** shows a *P* orientation for helices 3 and 4 in DCM (CD (±)), helix 2 rotates in the same *P* direction, whereas the internal helix 1 must describe the opposite rotation sense (*M* helix) according to the *cis–transoidal* configuration adopted by the polyene. Computational studies (TD-DFT(CAM-B3LYP)/3-21) performed on the *M* helix (*n* = 20) for poly-**3**, namely the *cis*–*transoidal* skeleton (ω_1_ = 175°), confirmed this hypothesis. To reduce the computational costs, the chiral moiety was removed from the pendants, keeping only the achiral spacer. This modification in the model allowed us to create a 3D structure describing a half helix turn, which is necessary to observe the external helices described by the OPEs (helix 3 and helix 4). The calculated ECD is in full agreement with the experimental one obtained in DCM, indicating that the proposed model is a good approximation to the highly stretched *cis*–*transoidal* structure adopted by the polymer.

The helical pitch value is in good agreement with the data extracted from the small-angle X-ray scattering (SAXS) measurements (4.0 nm) (see Figure S28). The combined information obtained from the ECD, SAXS, and AFM studies indicates that poly-**2** adopts a *cis–transoidal* scaffold (ω_1_ ca., 165°) where internal and external helices rotate in opposite directions (*P*_int_ (CD_405 nm_= (+)) and *M*_ext_ (AFM= (−)) (Figure 4b).

The 1D SAXS profile obtained for poly-**3** revealed two maxima. These diffraction peaks confirm the proposed 3D model for the polymer in which the 5.6 nm value is coincident with the helix width and the 17.0 nm is conincident with the helical pitch (see Figure S28). Further ECD studies indicated that the helical sense described by the OPE array in poly-**3** depends on the dielectric constant of the solvent (ε – 1)/(ε + 1), which denotes that poly-**3** is a dynamic helical polymer that acts as a chiroptical switch triggered by subtle variations of polarity. Thus, in solvents with (ε – 1)/(ε + 1) > 0.8 (i.e., ODCB, DCM, and 1,2-DCE), a bisignated (±) CD signature is induced in poly-**3**, which corresponds to a *P* helix of the OPE array (helices 3 and 4), namely the *M*_1_/*P*_3_/*P*_4_/*P*_2_ multihelix scaffold.

On the other hand, those solvents with (ε – 1)/(ε + 1) < 0.8 (i.e., CCl_4_, CHCl_3_, THF, and toluene) produce an *M* orientation of the OPE helical array (helices 3 and 4), yielding a *P*_1_/*M*_3_**/***M*_4_/*M*_2_ multihelix material (Figures S16c and d).

## Conclusions

**Figure 6:**
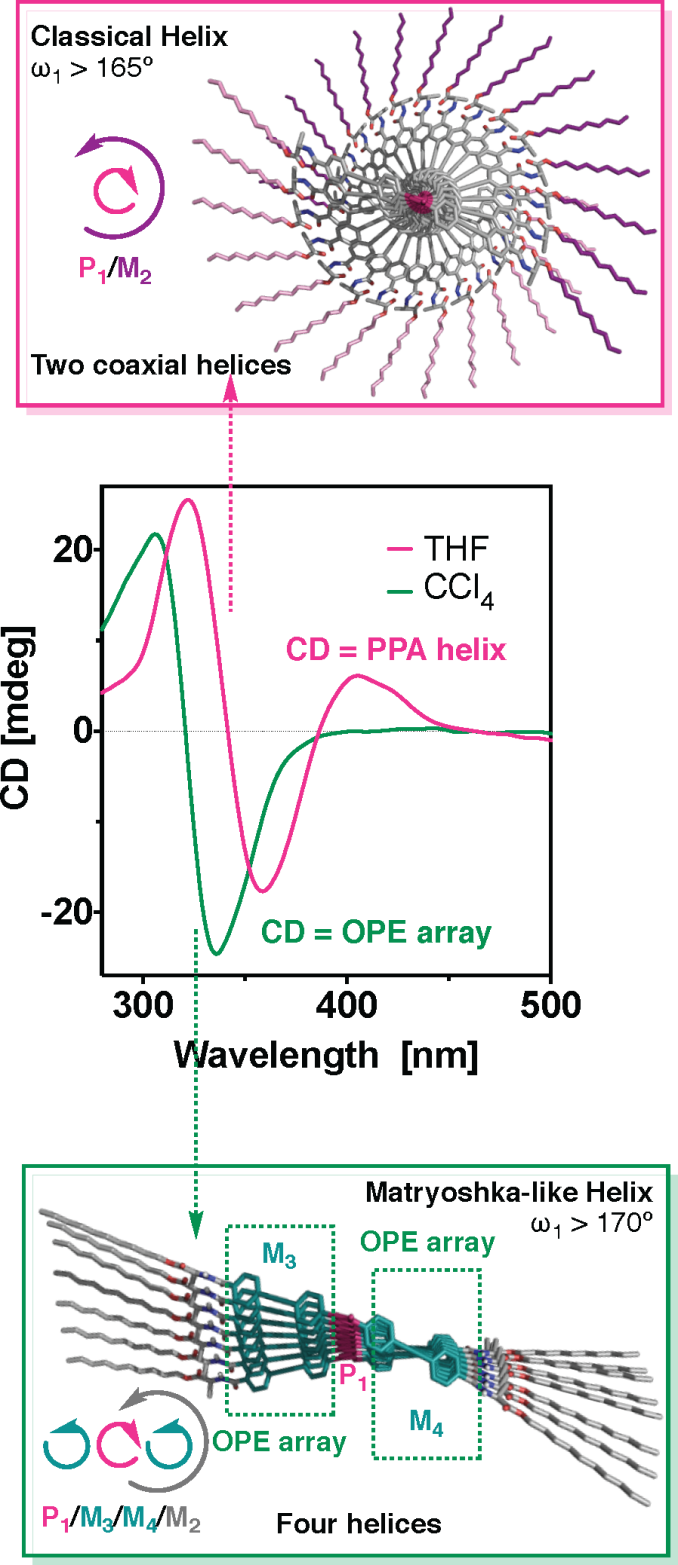
Moving from a classical helical PPA to a multihelix material by playing with the dihedral angle between conjugated double bonds.

In conclusion, we have demonstrated through two different examples, namely poly-**2** and poly-**3**, that it is possible to obtain a multihelix material by linking a supramolecular helix made by OPE units to a covalent helical polymer (PA). From previous studies in the fields of both covalent and supramolecular polymers, we envisioned that both structures could fit on a proper multihelix scaffold. In this special case of PA and OPE systems, the polyene should adopt an almost planar but twisted helical structure (i.e., a *cis–transoidal* helix with ω_1_ > 165°). This fact causes the pendant groups of the PA, in this case the OPE derivatives, to have a tilting degree between them (Θ) close to 11°, similar to that present in the supramolecular OPE polymers. In this way the multihelix material can be prepared by stabilizing a SP helix in a covalent helix. A perfect example is poly-**2**, which bears an OPE with *n* = 1 (Figure 6). In THF, this polymer a helical structure with ω_1_ ca. 165°, showing a CD signature with three alternating Cotton effects (a classical PPA helix). On the other hand, when solubilized in toluene or CCl_4_, poly-**2** adopts a more stretched helical scaffold with ω_1_ > 170°. This results in a bisignate CD signature that is governed by the axial orientation of the OPE units instead of being commanded by the helical orientation of the PA main chain. Consequently, two new helices emerge within this novel helical scaffold where four different helices coexist in the helical material: the two coaxial helices, namely internal (helix 1) and external (helix 2), and the two helices described by the OPE axial array (helices 3 and 4). These four helices are interconnected and by identifying the orientation of one of them it is possible to obtain the helical sense of the others. Therefore, two scenarios are possible: *M*_1_/*P*_3_/*P*_4_/*P*_2_ and *P*_1_/*M*_3_/*M*_4_/*M*_2_. For its part, the second design, poly-**3**, always generates a multihelix material due to the axial array of its OPE units, with the presence of four helices within a single polymer. These results open a new horizon in the design of helical polymers, where the stabilization of supramolecular helices in covalent polymers will allow the use of these structures in applications that previously could not be accessed due to the difficulty of generating SP polymers in polar solvents.

## Supporting Information Available

Materials and methods, synthesis of monomers and polymers, GPC studies, TGA studies, dynamic behavior, formation of SP-**3**, AFM measurements for poly-**2**, SAXS experiments, theoretical calculations, and supporting references (PDF)

## Abbreviations

PA: phenylacetylene
PPA: poly(phenylacetylene)
OPE: oligo(*p*-phenyleneethynylene)
ECD: electronic circular dichroism
*P*: plus
*M*: minus
UV: ultraviolet
POPEPA: poly[oligo(*p*-phenyleneethynylene)phenylacetylene]
Θ: tilting degree
DCM: dichloromethane
THF: tetrahydrofuran
DMF: *N*,*N*-dimethylformamide
AFM: atomic force microscopy
HOPG: highly-oriented pyrolytic graphite
ODCB: *ortho*-dichlorobenzene
1,2-DCE: 1,2-dichloroethane
fwhm: full-width at half-maximum
SAXS: small-angle X-ray scattering.
