# Supplementary material for: Merging Supramolecular and Covalent Helical Polymers: Four Helices Within a Single Scaffold
Source: J Am Chem Soc. 2021 Dec 3;143(49):20962–9. doi: 10.1021/jacs.1c10327 (PMC8679087; doi:10.1021/jacs.1c10327)
Supplement: Supplementary file 1 — ja1c10327_si_001.pdf [file ja1c10327_si_001.pdf]

Merging Supramolecular and Covalent Helical Polymers: Four Helices Within a Single Scaffold

Zulema Fernández,[a] Berta Fernández,[b] Emilio Quiñoá [a] and Félix Freire[*, a]

[a] Center for Research in Biological Chemistry and Molecular Materials (CiQUS) and Department of Organic Chemistry. University of Santiago de Compostela, 15782, Santiago de Compostela, Spain.

[b] Departamento de Química Física. University of Santiago de Compostela, 15782, Santiago de Compostela, Spain.

felix.freire@usc.es

http://felixfreire.com

Supporting Information

Contents

1. Materials and Methods 1

2. Synthesis of Monomers..2

3. Synthesis of Polymers..21

3.1 General Procedure for Polymerization 21

3.2 NMR Experiments..21

3.2.1 ^{1}H NMR Experiments for poly-**2** and poly-**3** 21

3.2.2 T_2 Experiments 23

3.2.3 STD NMR Experiments..24

3.3 Raman Experiments..28

4. GPC Studies 29

4.1 Polymers Stability Studied by GPC 29

5. TGA Studies 29

6. Dynamic Beahviour 30

6.1 Studies for poly-2 30

6.1.1 Analysis in Different Solvents..30

6.1.2 Raman Experiments for the Elongated and Stretched Scaffolds 30

6.1.3 Interaction with Metal Ions..31

6.2 Studies for poly-3 32

6.2.1 Analysis in Different Solvents..32

6.2.2 Interaction with Metal Ions and Effect of the Temperature..................................33

7. Formation of SP-3..34

8. Atomic Force Microscopy (AFM) Measurements for poly-2..............................35

9. SAXS Experiments 36

10. Theoretical Calculations 37

10. Supporting References 42

1. Materials and Methods

NMR experiments have been recorded in a Bruker spectromer (^{1}H frequency 300 mHz).

^{1}H NMR T_2 relaxation experiments and ^{1}H NMR STD experiments have been measured at 300 K in a Bruker NEO-750 spectrometer (^{1}H frequency 750 mHz).

CD measurements were done in a Jasco-720 and UV spectra were registered in a Jasco V-630. The amount of polymer used is indicated in the corresponding section.

VT-CD were measured in a Jasco-1100.

Raman spectra were carried out in a Reinshaw confocal Raman spectrometer (Invia Reflex model) with 785 nm diode laser.

GPC studies were carried out in a Waters Alliance equipped with Phenomenex GPC columns (10^3 Å, 10^4 Å and 10^5 Å). The amount of polymer used was 0.5 mg mL^{-1}. THF was used as eluent (flow rate: 1 mL min^{-1}) and as inner standard, polystyrene narrow standards (PSS) were used.

For the metal ions titrations, poly-**2** and poly-**3** were dissolved in $CHCl_3$ and the correspondent metal salt solution was then added. The amount of polymer used is indicated in the corresponding section.

TGA traces were obtained in a TGA Q5000 (TA Instruments, New Castle, UK) using a platinium pan.

SAXS experiments were performed in a SAXSPOINT 5.0 X-ray diffractometer. Primux 100 micro-Cu was the microfocus X-ray source with an ASTIX optics. The detector was an HPC Pilatus3 R aM model. The sample was monted in a plate for solid samples sealed with Kapton windows and placed at 1300 mm away from the detector.

PyMOL was used as a molecular visualization system.

Spartan 10 (MMFF94) was used for molecular modelling.

2. Synthesis of Monomers

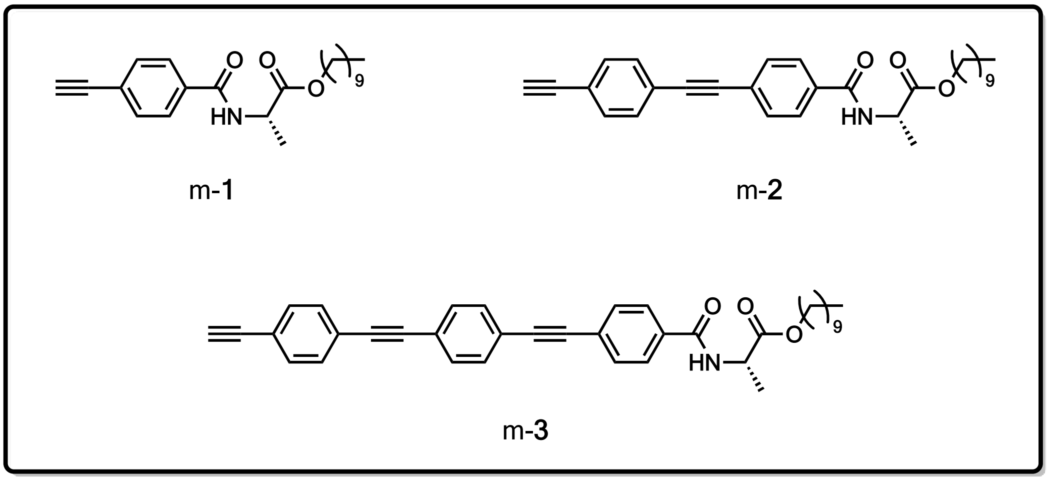

m-**1** was synthesized according to previously reported procedures.[S1]

Synthesis of heptyl-4-iodobenzoate (**1**)

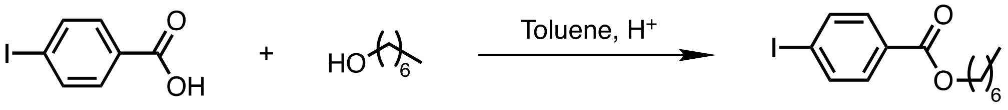

1

4-iodobenzoic acid (6.00 g, 24.19 mmol, 1 equiv), 1-heptanol (6.50 mL, 45.96 mmol, 1.90 equiv), toluene (50.00 mL) and a catalytic amount of H_2SO_4 (0.10 mL) were introduced in a round bottom flask fitted with a Dean-Stark. The mixture was heated at reflux temperature overnight. The organic phase was washed twice with Milli-Q water and brine. The crude product was chromatographed on silica gel (70-230 mesh) using hexane as eluent, obtaining **1** an orange oil (8.60 g, 97% yield).

^{1}H NMR (300 MHz, $CDCl_3$) δ_H (ppm): 7.79 (d, 2H, ArH), 7.73 (d, 2H, ArH), 4.29 (t, 2H, CH_2), 1.84-1.67 (m, 2H, CH_2), 1.50-1.17 (m, 8H, CH_2), 0.88 (t, 3H, CH_3).

^{13}C NMR (75 MHz, $CDCl_3$) δ_C (ppm): 166.1 (C=O), 137.6 (ArC), 131.0 (ArC), 130.0 (C_{sp}-Ar), 100.5 (C_{sp}-Ar), 65.4 (CH_2), 31.7 (CH_2), 28.9 (CH_2), 28.7 (CH_2), 26.0 (CH_2), 22.6 (CH_2), 14.1 (CH_3).

HRMS (APCI) m/z calcd for $C_{14}H_{19}IO_2$ $[M+H]^+$: 347.0430, found: 347.0500.

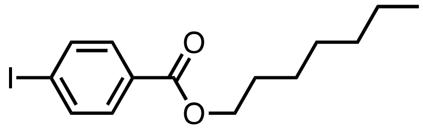

1

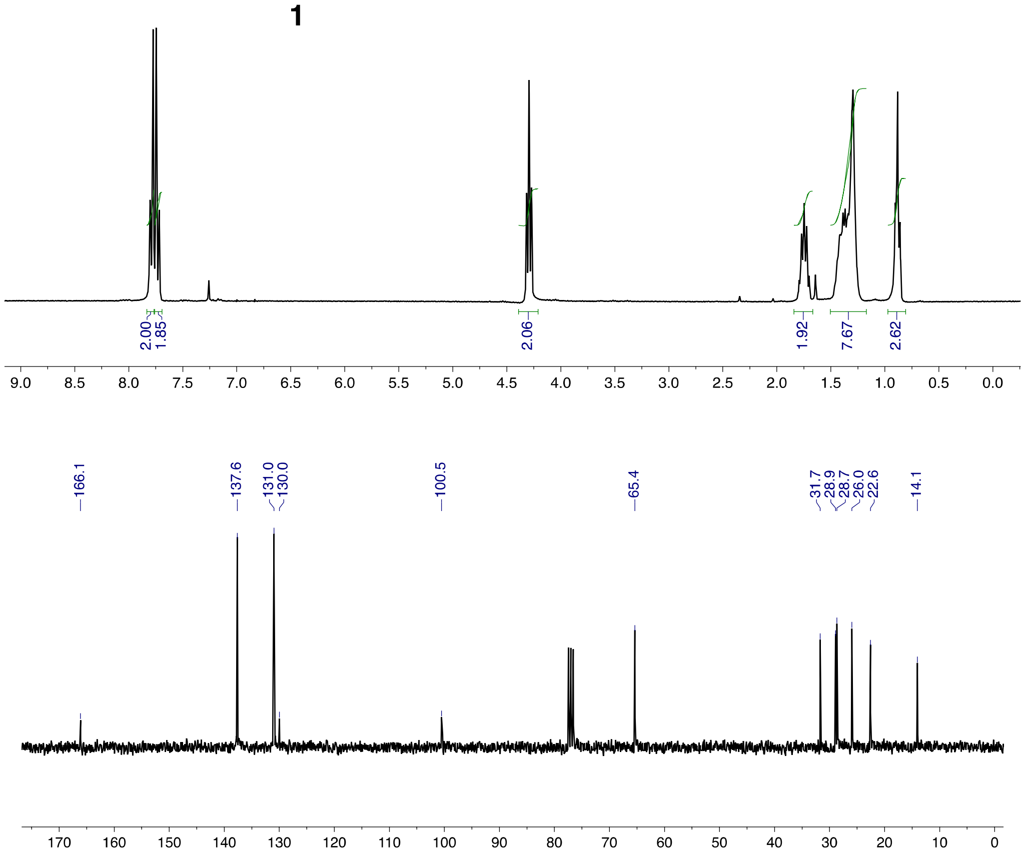

Figure S1. ^{1}H-NMR and ^{13}C-NMR of **1** in $CDCl_3$.

Syntesis of heptyl 4-((trimethylsilyl)ethynyl)benzoate (**2**)

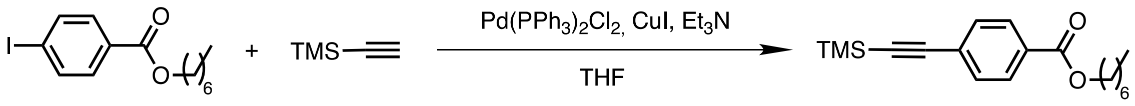

1 **2**

Heptyl-4-iodobenzoate (8.00 g, 23.10 mmol, 1.00 equiv), bis(triphenylphosphine)palladium(II) dichloride ($Pd(PPh_3)_2Cl_2$, 0.32 g, 0.46 mmol, 0.02 equiv) and copper iodide (CuI, 0.09 g, 0.46 mmol, 0.02 equiv) were dissolved in dry THF (50.00 mL). Next triethylamine (Et_3N, 15.00 mL) and ethynyltrimethylsilane (3.20 mL, 23.10 mmol, 1.00 equiv) were added and the mixture was stirred for three hours at room temperature. After removing the solvent, the crude product was chromatographed on silica gel (70-230 mesh) using hexane as eluent (7.00 g, 96% yield).

^{1}H NMR (300 MHz, $CDCl_3$) δ_H (ppm): 7.96 (d, 2H, ArH), 7.73 (d, 2H, ArH), 4.29 (t, 2H, CH_2), 1.84-1.66 (m, 2H, CH_2), 1.47-1.18 (m, 8H, CH_2), 0.88 (t, 3H, CH_3), 0.26 (s, 7H, $Si(CH_3)_3$).

^{13}C NMR (75 MHz, $CDCl_3$) δ_C (ppm): 166.0 (C=O), 131.8 (ArC), 130.1 (ArC), 129.3 (C_{sp}-ArC), 127.6 (C_{sp}-ArC), 104.1 (C_{sp}- AlkyneC), 97.5 (C_{sp}- AlkyneC), 65.3 (CH_2), 31.7 (CH_2), 28.9 (CH_2), 28.7 (CH_2), 26.0 (CH_2), 22.6 (CH_2), 14.0 (CH_3), -0.2 (TMS).

HRMS (APCI) m/z calcd for $C_{19}H_{29}O_2Si$ $[M+H]^+$: 317.1859, found: 317.1928.

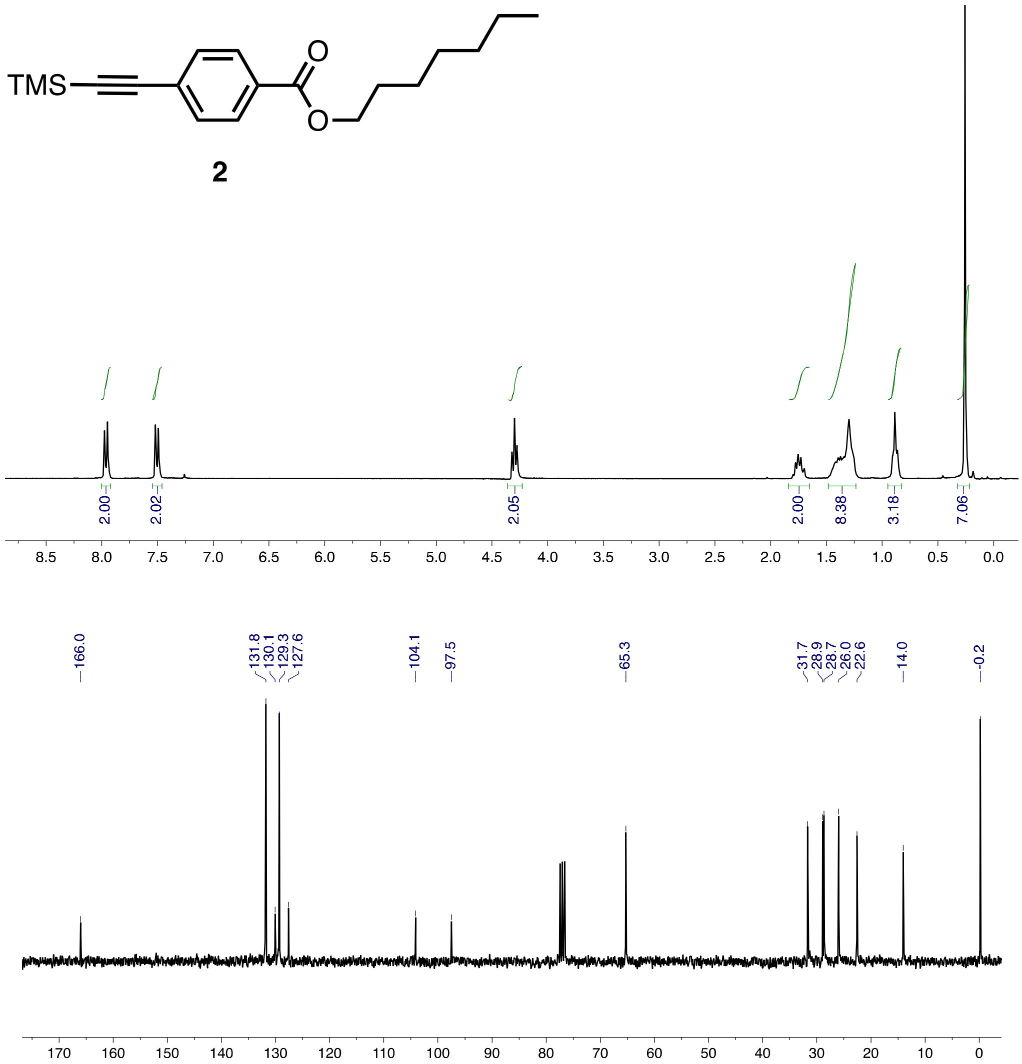

Figure S2. ^{1}H-NMR and ^{13}C-NMR of **2** in $CDCl_3$.

Synthesis of heptyl 4-ethynylbenzoate (**3**)

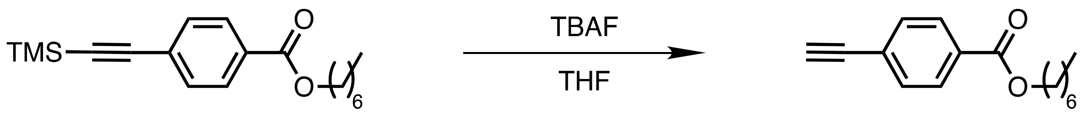

2 **3**

Heptyl 4-((trimethylsilyl)ethynyl)benzoate (7.00 g, 22.11 mmol, 1.00 equiv) was dissolved in THF (50 mL). Next TBAF (1M in hexane, 26.54 mL, 26,54 mmol, 1.20 equiv) was added and the reaction was stirred at r.t. for 15 minutes. After this time the organic solvent was removed and the crude was chromatographed on silica gel (70-230 mesh) with hexane/ethyl acetate (80:20) as eluent (4.21 g, 77% yield).

^{1}H NMR (300 MHz, $CDCl_3$) δ_H (ppm): 7.85 (d, 2H, ArH), 7.53 (d, 2H, ArH), 4.30 (t, 2H, CH_2), 3.22 (s, 1H, CH), 1.89-1.66 (m, 2H, CH_2), 1.51-1.20 (m, 8H, CH_2), 0.88 (t, 3H, CH_3).

^{13}C NMR (75 MHz, $CDCl_3$) δ_C (ppm): 165.9 (C=O), 132.0 (ArC), 130.5 (ArC), 129.4 (C_{sp}-ArC), 126.6 (C_{sp}-ArC), 82.8 (C_{sp}-AlkyneC), 79.9 (C_{sp}-AlkyneC), 65.4 (CH_2), 31.7 (CH_2), 28.9 (CH_2), 28.7 (CH_2), 26.0 (CH_2), 22.6 (CH_2), 14.0 (CH_3).

HRMS (APCI) m/z calcd for $C_{16}H_{22}O_2$ $[M+H]^+$: 245.1463, found: 245.1535.

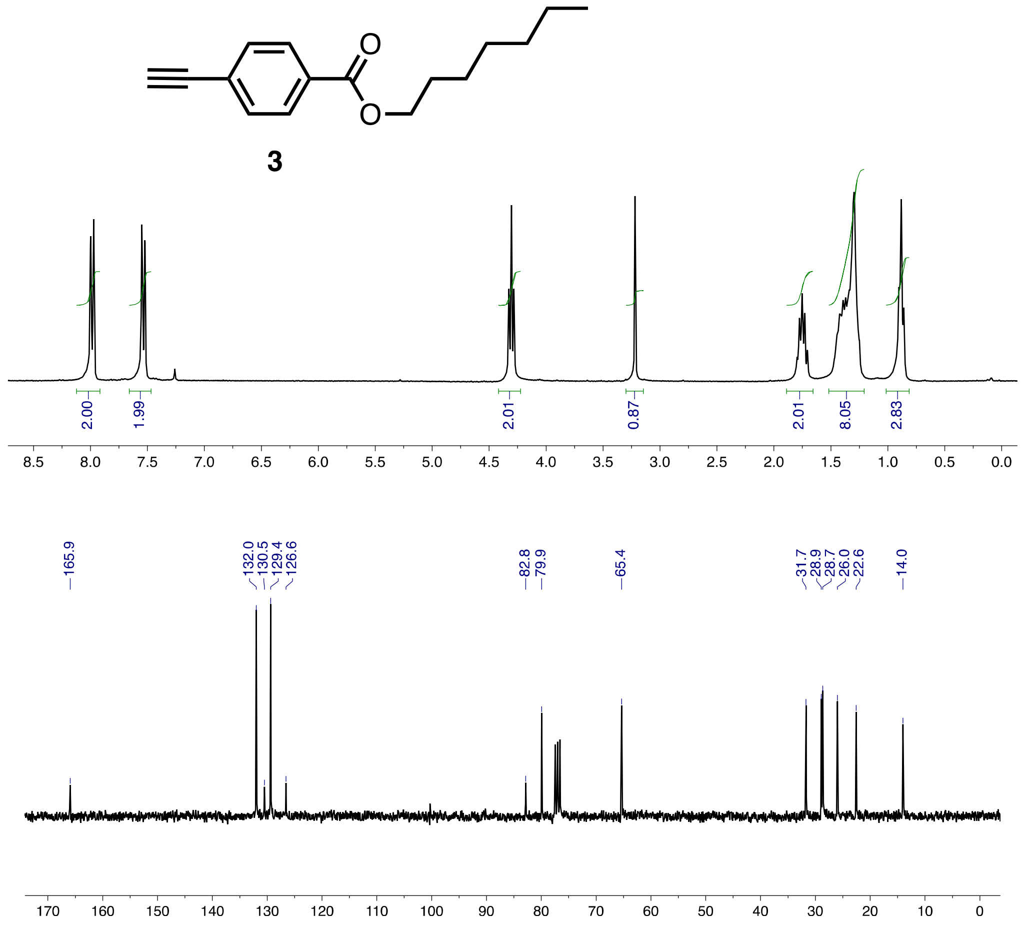

Figure S3. ^{1}H-NMR and ^{13}C-NMR of **3** in $CDCl_3$.

Synthesis of heptyl 4-((4-((trimethylsilyl)ethynyl)phenyl)ethynyl)benzoate (**4**)

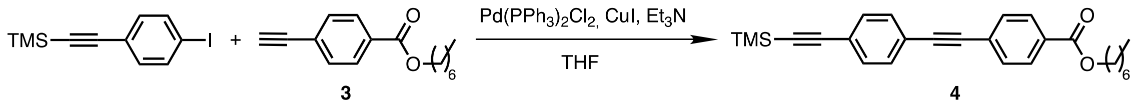

((4-iodophenyl)ethynyl)trimethylsilane (3.68 g, 12.27 mmol, 1.00 equiv), bis(triphenylphosphine)palladium(II) dichloride ($Pd(PPh_3)_4$, 0.85 g, 0.73 mmol, 0.02 equiv) and copper iodide (CuI, 0.14 g, 0.73 mmol, 0.02 equiv) were dissolved in dry Et_3N (40.00 mL). Next heptyl 4-ethynylbenzoate (3.68 g, 12.27 mmol, 1.00 equiv) was dissolved in $CHCl_3$ and added to the reaction. The mixture was stirred at 40 °C for three hours. After removing the solvent the crude product was chromatographed on silica gel (70-230 mesh) with hexane/dichloromethane (90:10) as eluent (4.02 g, 78% yield).

^{1}H NMR (300 MHz, $CDCl_3$) δ_H (ppm): 8.02 (d, 2H, ArH), 7.57 (d, 2H, ArH), 7.46 (s, 4H, ArH), 4.32 (t, 2H, CH_2), 1.84-1.65 (m, 2H, CH_2), 1.50-1.18 (m, 8H, CH_2), 0.90 (t, 3H, CH_3), 0.25 (s, 7H, TMS).

^{13}C NMR (75 MHz, $CDCl_3$) δ_C (ppm): 166.0 (C=O), 131.9 (ArC), 131.5 (ArC), 131.5 (ArC), 130.0 (C_{sp}-ArC), 129.5 (ArC), 127.5 (C_{sp}-ArC), 123.4 (C_{sp}-ArC), 122.7 (C_{sp}-ArC), 104.5 (C_{sp}- AlkyneC), 96.6 (C_{sp}- AlkyneC), 91.8 (C_{sp}- AlkyneC), 90.5 (C_{sp}- AlkyneC), 65.3 (CH_2), 31.7 (CH_2), 28.9 (CH_2), 28.7 (CH_2), 26.0 (CH_2), 22.6 (CH_2), 14.1 (CH_3), -0.1 (TMS).

HRMS (ESI) m/z calcd for $C_{27}H_{32}O_2Si$ $[M+H]^+$: 417.2172, found: 417.2242.

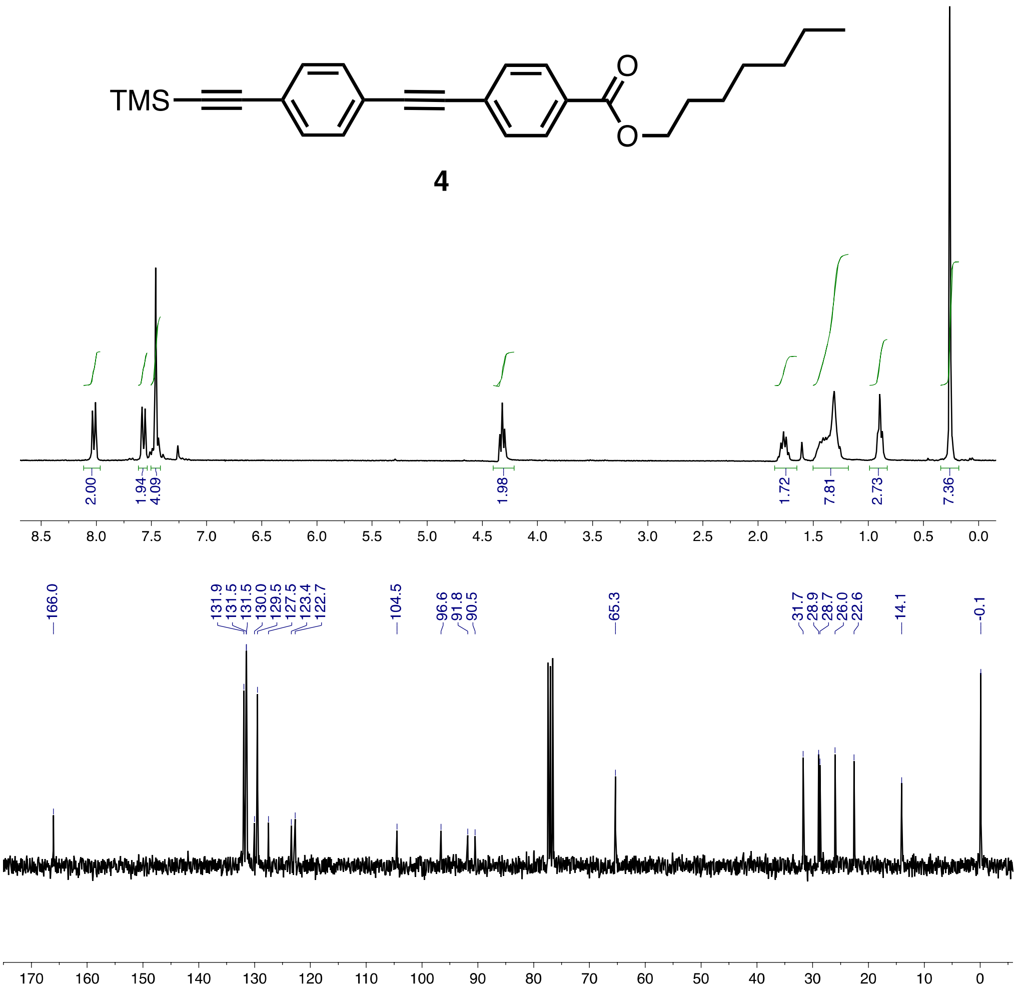

Figure S4. ^{1}H-NMR and ^{13}C-NMR of **4** in $CDCl_3$.

Synthesis of 4-((4-ethynylphenyl)ethynyl)benzoic acid (**5**)

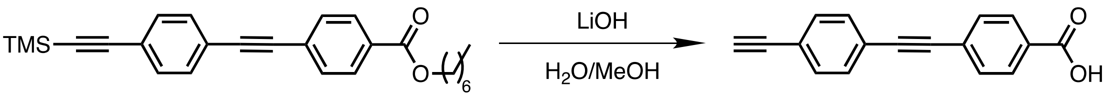

4 5

Heptyl 4-((4-((trimethylsilyl)ethynyl)phenyl)ethynyl)benzoate (4.00 g, 9.60 mmol, 1.00 equiv) was dissolved in MeOH (50 mL). Then Milli-Q water (30 mL) and LiOH (1.15 g, 48.00 mmol, 5.00 equiv) were added to the solution and the mixture was stirred at 60 °C. Upon completion of the reaction the solvent was removed under reduced preassure. The residue was redisolved in a small amount of water and acidified to pH= 1. After extracting with ethyl acetate (4 times), the solution was dried over Na_2SO_4. Next, the organic solvent was removed obtaining the product as an orange solid (2.00 g, 85% yield).

^{1}H NMR (300 MHz, DMSO) δ_H (ppm): 13.18 (s, 1H, COOH), 7.96 (d, 2H, ArH), 7.66 (d, 2H, ArH), 7.58 (d, 2H, ArH), 7.52 (d, 2H, ArH), 4.37 (s, 1H, CH).

^{13}C NMR (75 MHz, DMSO) δ_C (ppm): 171.8 (C=O), 137.3 (ArC), 137.0 (ArC), 136.8 (ArC), 136.0 (C_{sp}-ArC), 134.8 (ArC), 131.4 (C_{sp}-ArC), 127.6 (C_{sp}-ArC), 127.4 (C_{sp}-ArC), 96.4 (C_{sp}- AlkyneC), 95.7 (C_{sp}- AlkyneC), 88.3 (C_{sp}- AlkyneC, 2 positions).

HRMS (ESI) m/z calcd for $C_{17}H_{10}O_2$ $[M+H]^+$: 247.0681 , found: 247.0752.

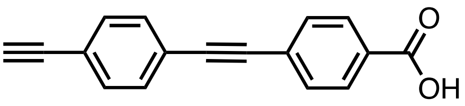

5

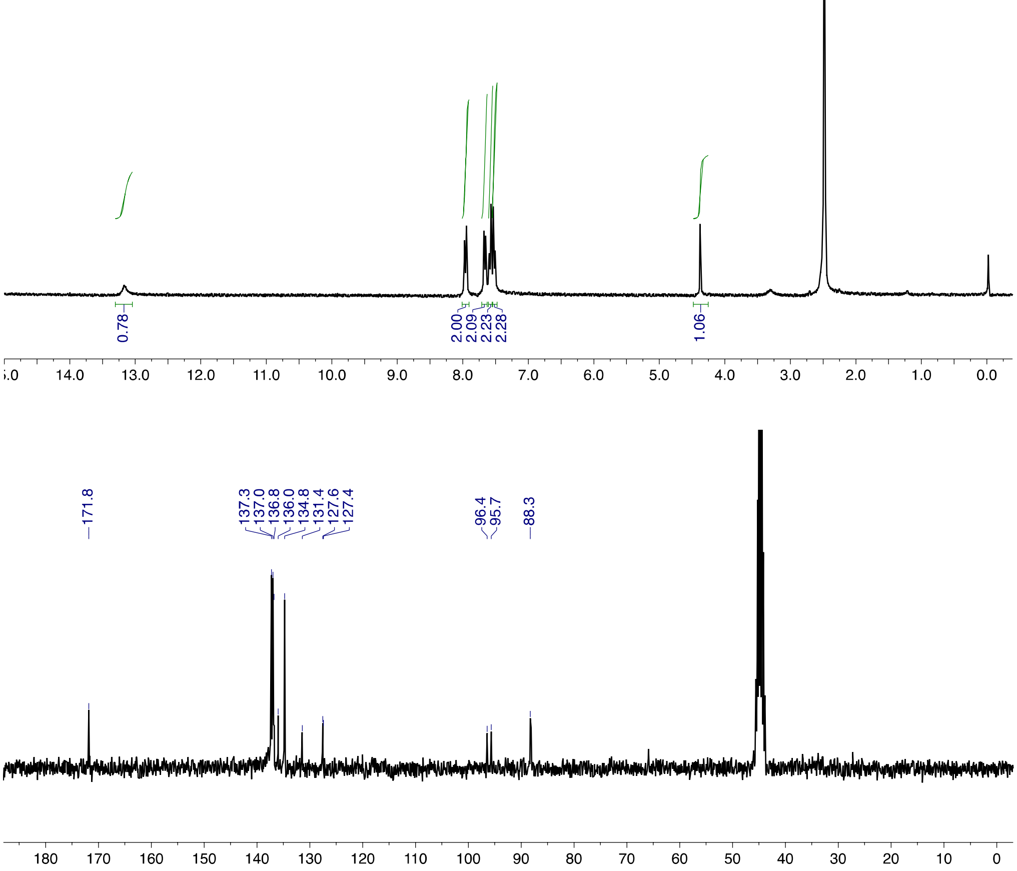

Figure S5. ^{1}H-NMR and ^{13}C-NMR of **5** in DMSO-d_6.

Synthesis of decyl (*tert*-butoxycarbonyl)-*L*-alaninate (**6**)

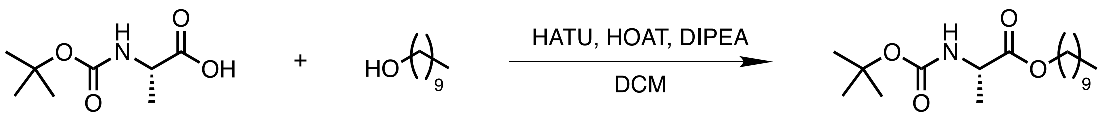

6

N-(tert-Butoxycarbonyl)-*L*-alanine (2.00 g, 10.60 mmol, 1.00 equiv), 2-(7-Aza-1H-benzotriazole-1-yl)-1,1,3,3-tetramethyluronium (HATU, 4.22 g, 11.10 mmol, 1.05 equiv), 1-hydroxy-7-azabenzotriazole (HOAt, 1.51 g, 11.10 mmol, 1.05 equiv) and diisopropyltriethylamine (DIEA, 2.22 mL, 12.68 mmol, 1.20 equiv) were dissolved in 50 mL of dry CH_2Cl_2. After 15 minutes, time needed to activate the acid, 1-decanol (2.00 mL, 10.60 mmol, 1.00 equiv) was added and the mixture was stirred overnight.. The crude product was chromatographed on silica gel (70-230 mesh) with hexane/ethyl acetate (90:10) as eluent (1.48 g, 43% yield).

^{1}H NMR (300 MHz, $CDCl_3$) δ_H (ppm): 5.11 (s, 1H, NH), 4.25 (s, 1H, CH), 4.08 (s, 2H, CH_2), 2.20 (s, 2H, CH_2), 1.65-1.52 (m, 2H, CH_2), 1.48-1.16 (m, 26H, CH_2, Boc and CH_3), 0.98-0.76 (t, 3H, CH_3).

^{13}C NMR (75 MHz, $CDCl_3$) δ_C (ppm): 173.4 (C=O), 155.0 (C=O), 79.6 (C_{sp}-Boc), 65.4 (CH_2), 49.2 (CH), 31.8 (CH_3), 29.4 (CH_2, 2 positions), 29.2 (CH_2), 29.1 (CH_2), 28.5 (CH_2), 28.3 (CH_3-Boc, 3 positions), 25.7 (CH_2), 22.6 (CH_2), 18.7 (CH_2), 14.0 (CH_3).

HRMS (ESI) m/z calcd for $C_{18}H_{36}NO_4$ $[M+H]^+$: 330.2566, found: 330.2638; calcd for $C_{18}H_{35}NO_3Na$ $[M+Na]^+$: 352.2566, found: 352.2462.

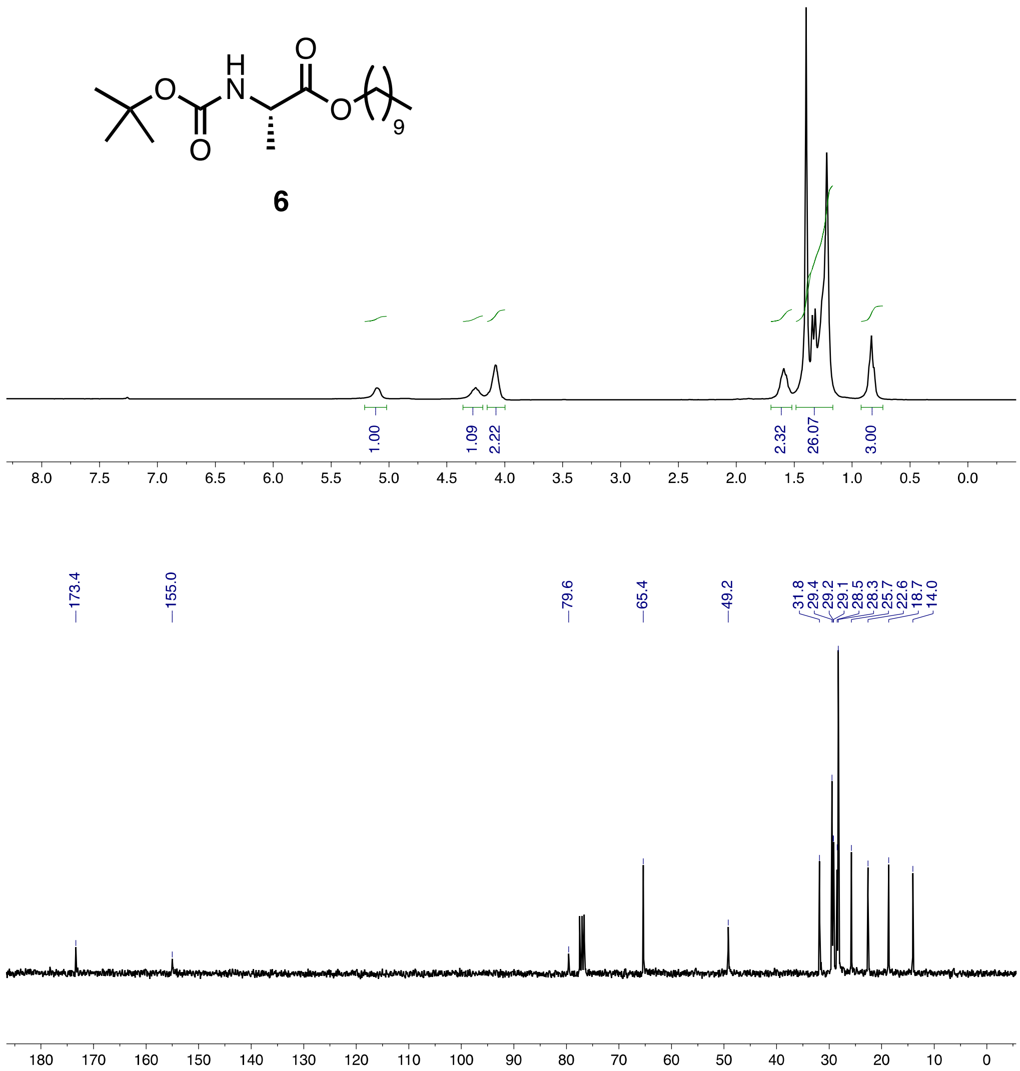

Figure S6. ^{1}H-NMR and ^{13}C-NMR of **6** in $CDCl_3$.

Synthesis of decyl (4-((4-ethynylphenyl)ethynyl)benzoyl)-*L*-alaninate (m-**2**)

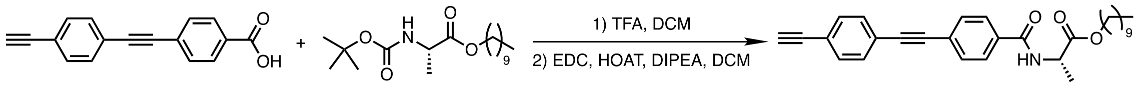

5 **6** m-**2**

Decyl (*tert*-butoxycarbonyl)-*L*-alaninate was disolved in DCM:TFA (7:3). After the completion of the reaction, the solvent was removed and the crude was washed twice with MeCN. Decyl *L*-alaninate was obtained as a colourless liquid.

4-((4-ethynylphenyl)ethynyl)benzoic acid (0.70 g, 2.84 mmol, 1.00 equiv), *N*-(3-Dimethylaminopropyl)-*N*′-ethylcarbodiimide hydrochloride (EDC, 0.57 g, 2.98 mmol, 1.05 equiv), 1-hydroxy-7-azabenzotriazole (HOAt, 0.41 g, 2.98 mmol, 1.05 equiv) and diisopropyltriethylamine (DIEA, 0.60 mL, 3.41 mmol, 1.20 equiv) were dissolved in 30 mL of dry CH_2Cl_2. After 15 minutes, time needed to activate the acid, decyl (*L*)-alaninate (0.94 g, 2.84 mmol, 1.00 equiv) was added and the mixture was stirred overnight. The organic layer was washed three times with HCl 1M, a saturated solution of $NaHCO_3$ and brine. The combined organic layers were dried over anhydrous Na_2SO_4, filtered and evaporated at reduced pressure. The crude product was chromatographed on silica gel (70-230 mesh) with hexane/ethyl acetate (80:20) as eluent (0.25 g, 78% yield).

^{1}H NMR (300 MHz, $CDCl_3$) δ_H (ppm): 7.82 (d, 2H, ArH), 7.59 (d, 2H, ArH), 7.50 (s, 4H, ArH), 6.86 (d, 1H, NH), 4.88-4.73 (q, 1H, CH), 4.26-4.14 (m, 2H, CH_2), 3.20 (s, 1H, CH), 1.78-1.63 (m, 2H, CH_2), 1.54 (d, 3H, CH_3), 1.42-1.24 (m, 14H, CH_2), 0.93-0.85 (t, 3H, CH_3).

^{13}C NMR (75 MHz, $CDCl_3$) δ_C (ppm): 173.3 (C=O), 166.0 (C=O), 133.4 (C_{sp}-ArC), 132.1 (ArC, 2 positions), 131.7 (ArC, 2 positions), 131.6 (ArC, 2 positions), 127.1 (ArC, 2 positions), 126.4 (C_{sp}-ArC), 123.2 (C_{sp}-ArC), 122.3 (C_{sp}-ArC), 91.2 (C_{sp}- AlkyneC), 90.5 (C_{sp}- AlkyneC), 83.2 (C_{sp}-AlkyneC), 79.3 (C_{sp}- AlkyneC), 65.8 (CH_2), 48.7 (CH), 31.9 (CH_3), 29.5 (CH_2, 2 positions), 29.3 (CH_2), 29.2 (CH_2), 28.5 (CH_2), 25.8 (CH_2), 22.7 (CH_2), 18.6 (CH_2), 14.1 (CH_3).

HRMS (ESI) m/z calcd for $C_{30}H_{35}NO_3$ $[M+H]^+$: 458.2717, found: 458.2688.

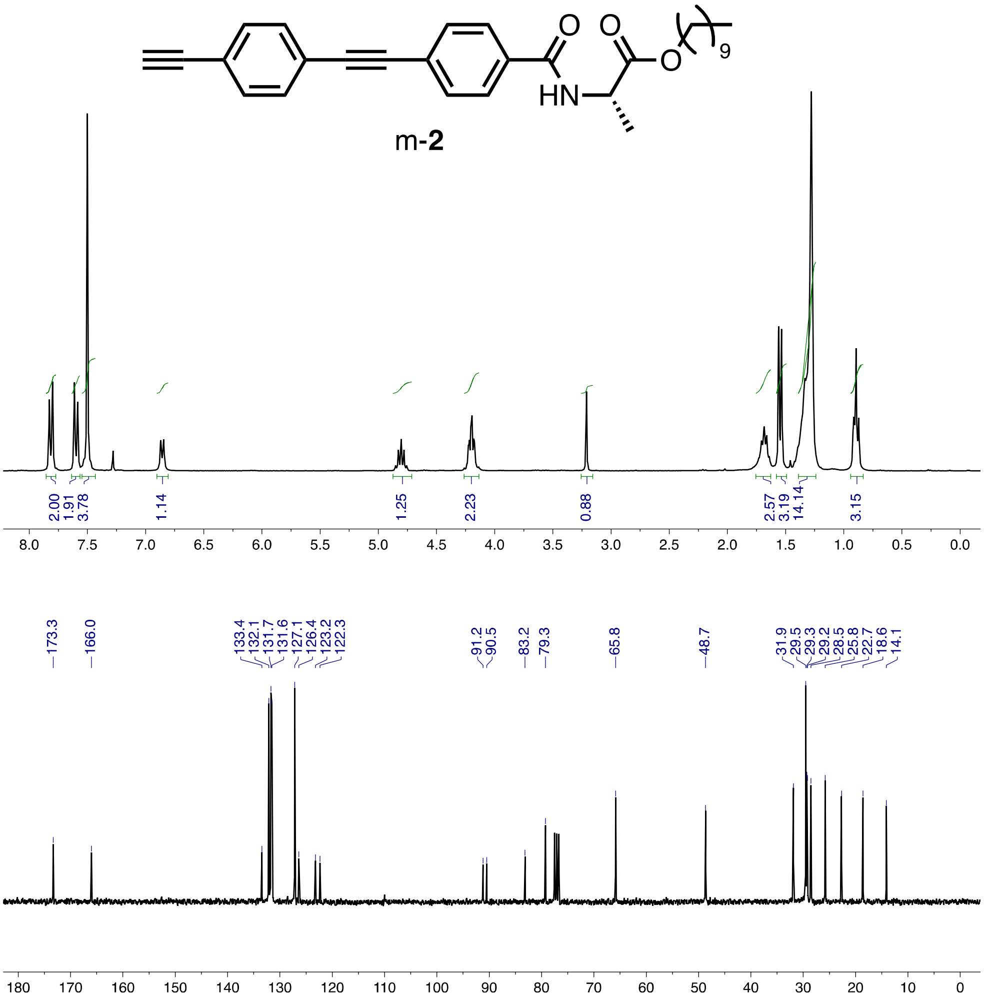

Figure S7. ^{1}H-NMR and ^{13}C-NMR of m-**2** in $CDCl_3$.

Synthesis of decyl (4-((4-((4-((trimethylsilyl)ethynyl)phenyl)ethynyl)phenyl)ethynyl)benzoyl)-*L*-alaninate (**7**)

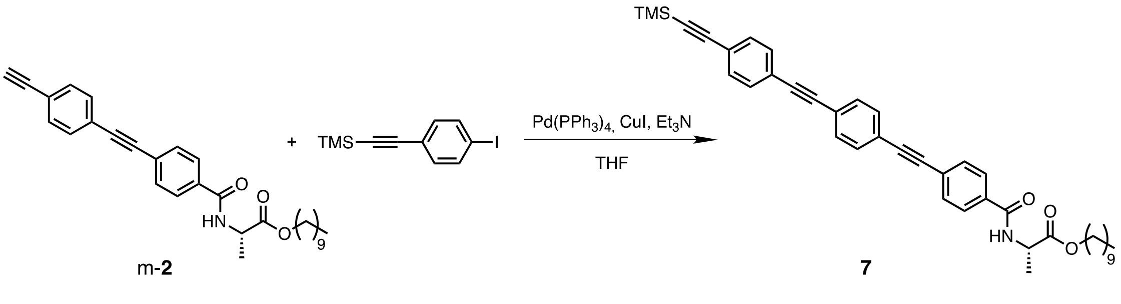

((4-iodophenyl)ethynyl)trimethylsilane (0.13 g, 0.44 mmol, 1.00 equiv), tetrakis(triphenylphosphine)palladium(0) ($Pd(PPh_3)_4$, 0.01 g, 0.01 mmol, 0.02 equiv) and copper iodide (CuI, 0.01 g, 0.01 mmol, 0.02 equiv) were dissolved in dry THF (10 mL). Next triethylamine (Et_3N, 5 mL) and decyl (4-((4-ethynylphenyl)ethynyl)benzoyl)-*L*-alaninate (**7**, 0.20 g, 0.44 mmol, 1.00 equiv) were added and the mixture was stirred at 40 °C overnight. After removing the solvent the crude product was chromatographed on silica gel (70-230 mesh) with hexane/ethyl acetate (60:40) as eluent (0.17 g, 63% yield).

^{1}H NMR (300 MHz, $CDCl_3$) δ_H (ppm): 7.79 (d, 2H, ArH), 7.56 (d, 2H, ArH), 7.50 (s, 4H, ArH), 7.44 (s, 4H, ArH), 6.89 (d, 1H, NH), 4.84-4.70 (q, 1H, CH), 4.23-4.12 (m, 2H, CH_2), 1.78-1.60 (m, 2H, CH_2), 1.52 (d, 3H, CH_3), 1.39-1.19 (m, 14H, CH_2), 0.98-0.79 (m, 3H, CH_3), 0.25 (s, 7H, TMS).

^{13}C NMR (75 MHz, $CDCl_3$) δ_C (ppm): 173.3 (C=O), 166.0 (C=O), 133.4 (C_{sp}-ArC), 131.9 (ArC, 2 positions), 131.7 (ArC, 2 positions), 131.7 (ArC, 2 positions), 131.6 (ArC, 2 positions), 131.4 (ArC, 2 positions), 127.1 (ArC, 2 positions), 126.5 (C_{sp}-ArC), 123.2 (C_{sp}-ArC), 123.2 (C_{sp}-ArC), 123.0 (C_{sp}-ArC), 122.7 (C_{sp}-ArC), 96.5 (C_{sp}- AlkyneC), 91.4 (C_{sp}- AlkyneC), 91.1 (C_{sp}- AlkyneC), 90.9 (C_{sp}- AlkyneC), 90.5 (C_{sp}- AlkyneC), 65.8 (CH_2), 48.6 (CH), 31.9 (CH_3), 29.5 (CH_2), 29.3 (CH_2), 29.2 (CH_2, 2 positions), 28.5 (CH_2), 25.8 (CH_2), 22.7 (CH_2), 18.7 (CH_2), 14.1 (CH_3), 0 (TMS).

HRMS (ESI) m/z calcd for $C_{41}H_{47}NO_3Si$ $[M+H]^+$: 630.3325, found: 630.3399.

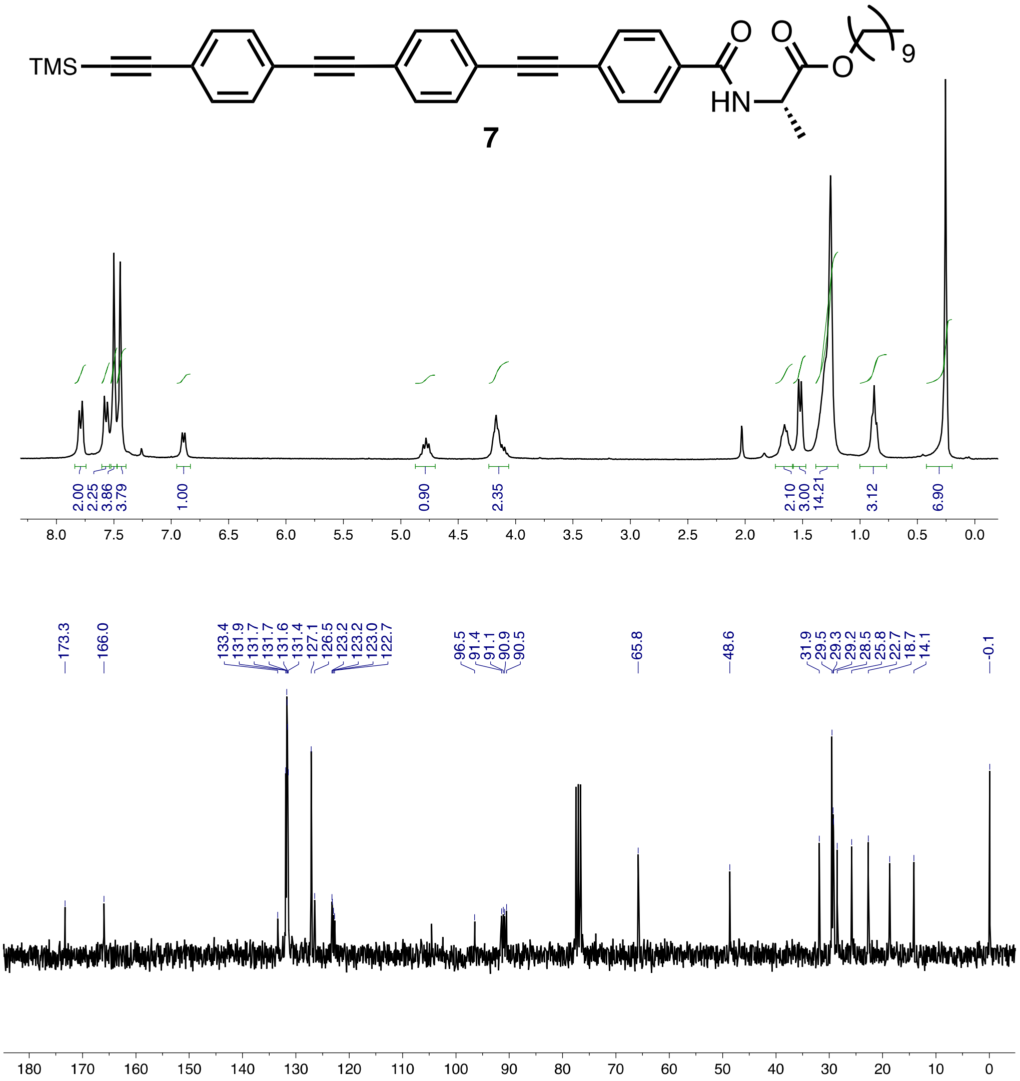

Figure S8. ^{1}H-NMR and ^{13}C-NMR of **7** in $CDCl_3$.

Synthesis of decyl (4-((4-((4-ethynylphenyl)ethynyl)phenyl)ethynyl)benzoyl)-*L*-alaninate (m-**3**)

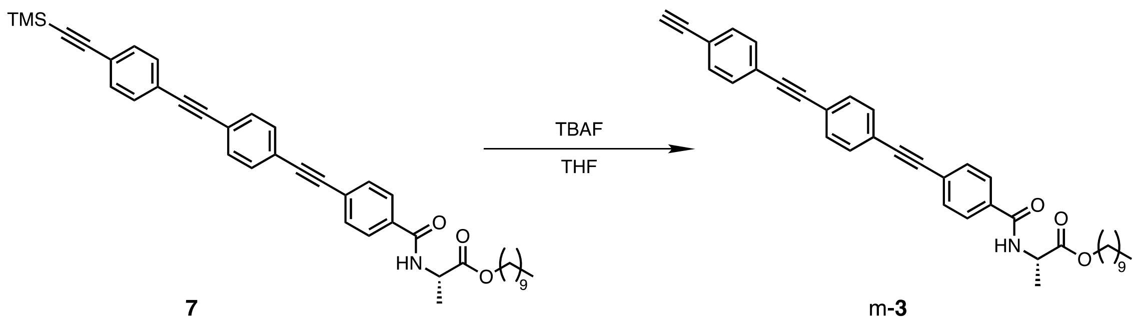

Decyl (4-((4-((4-((trimethylsilyl)ethynyl)phenyl)ethynyl)phenyl)ethynyl)benzoyl)-*L*-alaninate (**8**, 0.17 g, 0.27 mmol, 1.00 equiv) was dissolved in THF (5 mL). Next TBAF was added (1M in hexane, 0.32 mL, 0.32 mmol, 1.20 equiv) and the reaction was stirred at r.t. for 15 minutes. The crude was chromatographed on silica gel (70-230 mesh) with chloroform/ethyl acetate (90:10) as eluent (0.10 g, 70% yield).

^{1}H NMR (300 MHz, $CDCl_3$) δ_H (ppm): 7.80 (d, 2H, ArH), 7.58 (d, 2H, ArH), 7.52 (s, 4H, ArH), 7.74 (s, 4H, ArH), 6.83 (d, 1H, NH), 4.85-4.75 (q, 1H, CH), 4.24-4.12 (m, 2H, CH_2), 3.18 (s, 1H, CH), 1.74-1.62 (m, 2H, CH_2), 1.54 (d, 3H, CH_3), 1.36-1.23 (m, 14H, CH_2), 0.92-0.83 (t, 3H, CH_3).

^{13}C NMR (75 MHz, $CDCl_3$) δ_C (ppm): 173.3 (C=O), 165.9 (C=O), 133.5 (C_{sp}-ArC), 132.1 (ArC, 2 positions), 131.7 (ArC, 2 positions), 131.7 (ArC, 2 positions), 131.6 (ArC, 2 positions), 131.5 (ArC, 2 positions), 127.1 (ArC, 2 positions), 126.5 (C_{sp}-ArC), 123.4 (C_{sp}-ArC), 123.2 (C_{sp}-ArC), 122.8 (C_{sp}-ArC), 122.2 (C_{sp}-ArC), 91.4 (C_{sp}- AlkyneC), 90.9 (C_{sp}- AlkyneC), 90.5 (C_{sp}- AlkyneC), 87.5 (C_{sp}- AlkyneC), 83.2 (C_{sp}- AlkyneC), 79.1 (C_{sp}- AlkyneC), 65.9 (CH_2), 48.6 (CH), 31.9 (CH_3), 29.5 (CH_2, 2 positions), 29.3 (CH_2), 29.2 (CH_2), 28.5 (CH_2), 25.8 (CH_2), 22.7 (CH_2), 18.7 (CH_2), 14.1 (CH_3).

HRMS (APCI) m/z calcd for $C_{29}H_{23}NO_3$ $[M+H]^+$: 558.2930, found: 558.3005.

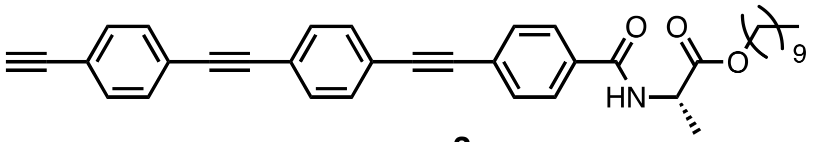

m-**3**

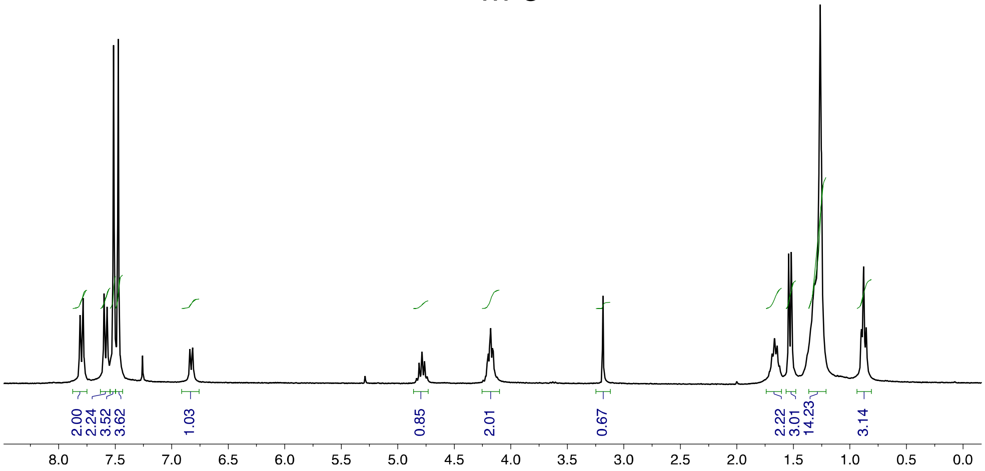

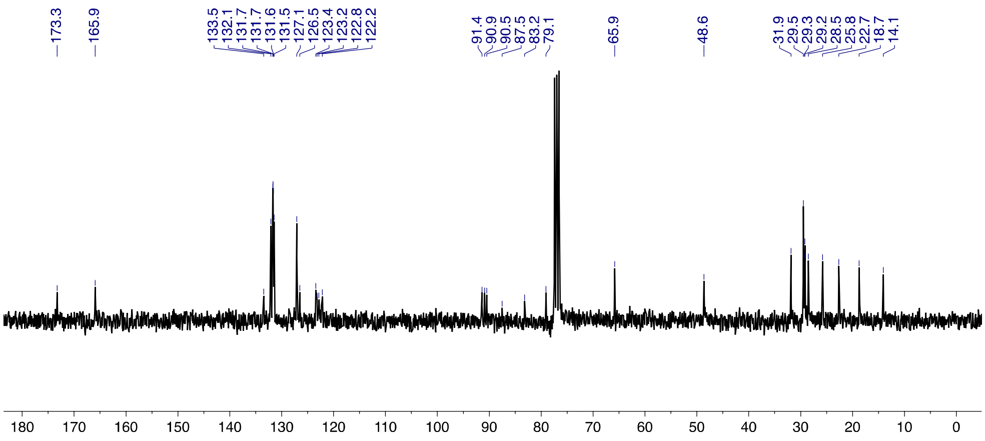

Figure S9. ^{1}H-NMR and ^{13}C-NMR of m-**3** in $CDCl_3$.

3. Synthesis of Polymers

3.1 General Procedure for Polymerization

The polymers were synthesized in a flask (sealed ampoule) previously dried under vacuum and flushed with Ar for three times. The monomers were added as a solid and dissolved in dry THF. Next a solution of rhodium norbadiene chloride dimer $[Rh(nbd)Cl]_2$ was added and the mixture was stirred overnight. The resulting polymers were diluted in DCM and precipitated in a large amount of MeOH, centrifuged (twice) and reprecipitated in hexane and centrifuged again.

Table S1. Calculated amounts for the polymers synthesis.

Monomer	Mass (mg)	THF (μL)	Et_3N (μL)	Catalyst (mg)	Yield (%)
poly-**2**	50	218	10	0.5	89
poly-**3**	50	300	10	0.4	91

3.2 NMR Experiments

3.2.1 ^1H NMR Experiments for poly-**2** and poly-**3**

^1H NMR spectroscopy in $CDCl_3$ indicates that poly-**2** adopts a *cis-* configuration in the polyene backbone (vinylic proton, δ = 5.7-5.8 ppm). However, integration of the peak signals do not match with the expected value. This is related to the difference in mobility of the protons within the molecule —a highly flexible chiral moiety and a more rigid core made up of the OPE units and the polyene backbone— that will need a different relaxation times for the correct signal integration.

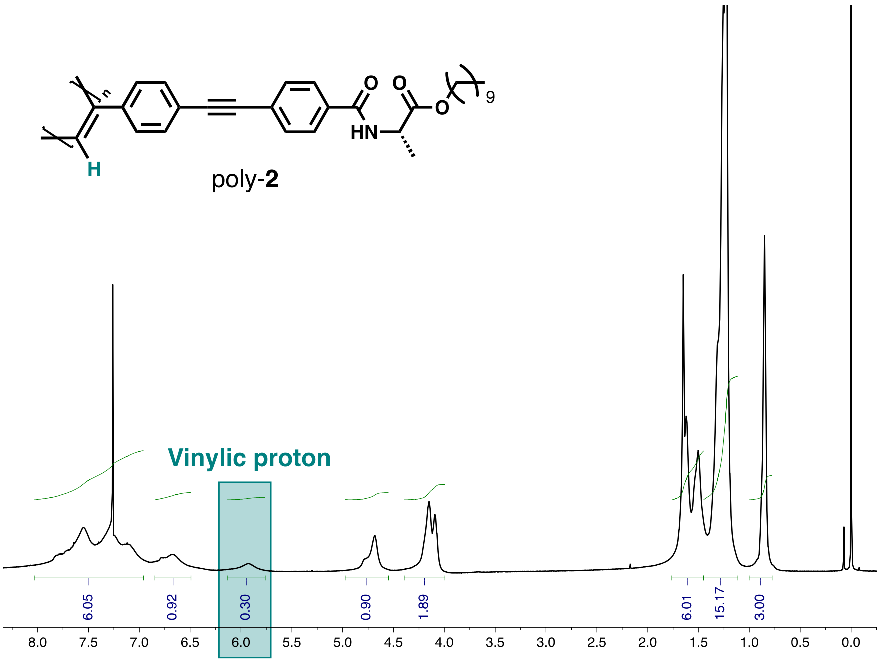

Figure S10. ^{1}H NMR for poly-**2** in $CDCl_3$.

A more complex situation is occurred when the ^{1}H NMR for poly-**2** is recoreded in CCl_4. In this case, not only the integration area of the peak do not match with the expected value, but also the highly stretched scaffold adopted by the polymer in solution results in a broadening of the signals bacause of its large aggregation tendency. Interestingly, in these conditions, the vinylic proton is not observed.

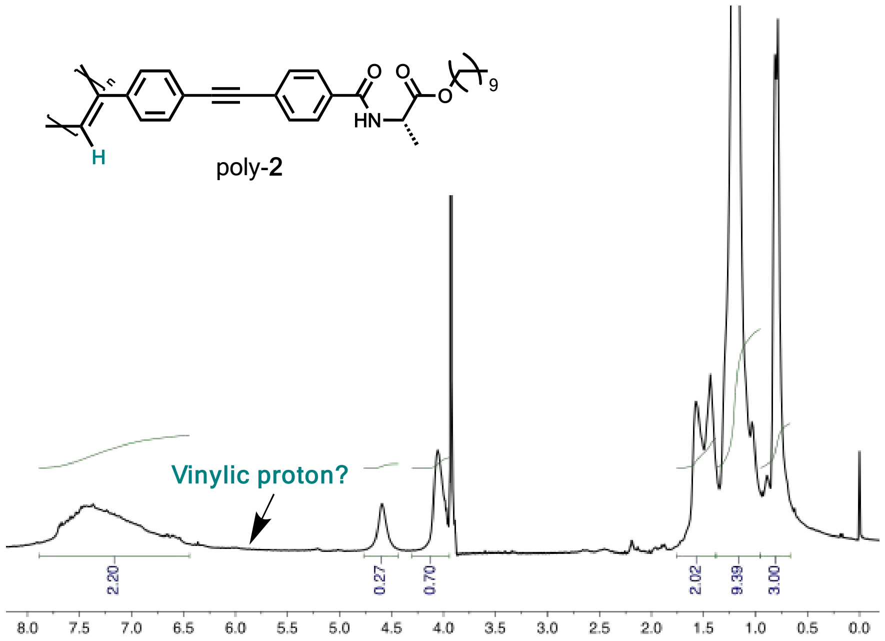

Figure S11. ^{1}H NMR for poly-**2** in CCl_4.

The dissapereance of the vinylic peak is confirmed by registering the ^{1}H NMR spectra of poly-**2** at different $CDCl_3$/CCl_4 ratios. By increasing the CCl_4 fraction, the vinyl signal at 5.93 ppm (100% $CDCl_3$) is shifted to 6.02 ppm (75% to 25% $CDCl_3$) and becomes null when no ammount of $CDCl_3$ is present (Figure S12).

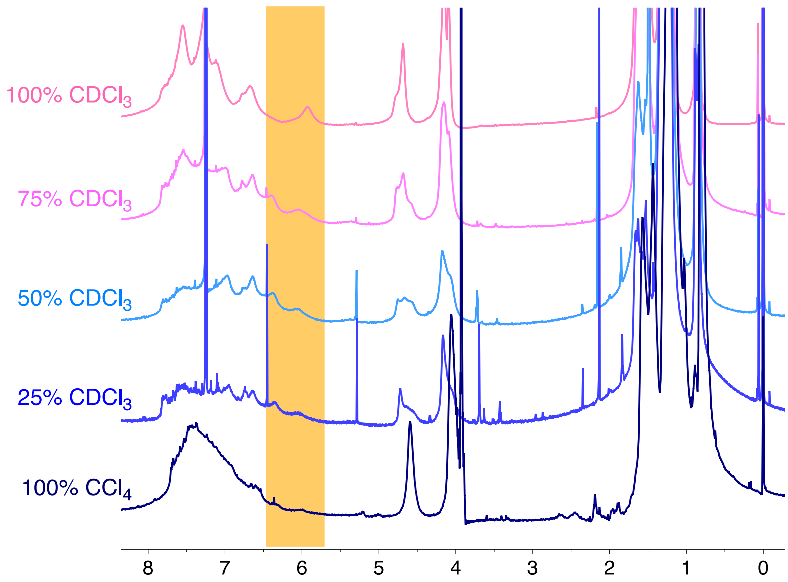

Figure S12. ^{1}H NMR comparision for poly-**2** with increasing ammounts of CCl_4.

This finding clearly indicates that the vanishing of this signal is directly related to the scaffold adopted by poly-**2** in solution. The structure displayed by the polymer will have a large impact on the polymer mobility, affecting directly to the relaxation time during NMR measurements and resuting in a broadening or dissapereance of NMR peaks.

A similar scenario to that observed for poly-**2** in CCl_4 occurs for poly-**3**. This polymer adopts a highly stretched and rigid scaffold in all the tested solvents and, as a consequence, no signal in the vinylic region is observed when registering the NMR spectra. As occurred before, the relaxation time during the NMR experiments will be affected by the high rigidity of the polymer scaffold, affecting to the peak integration area.

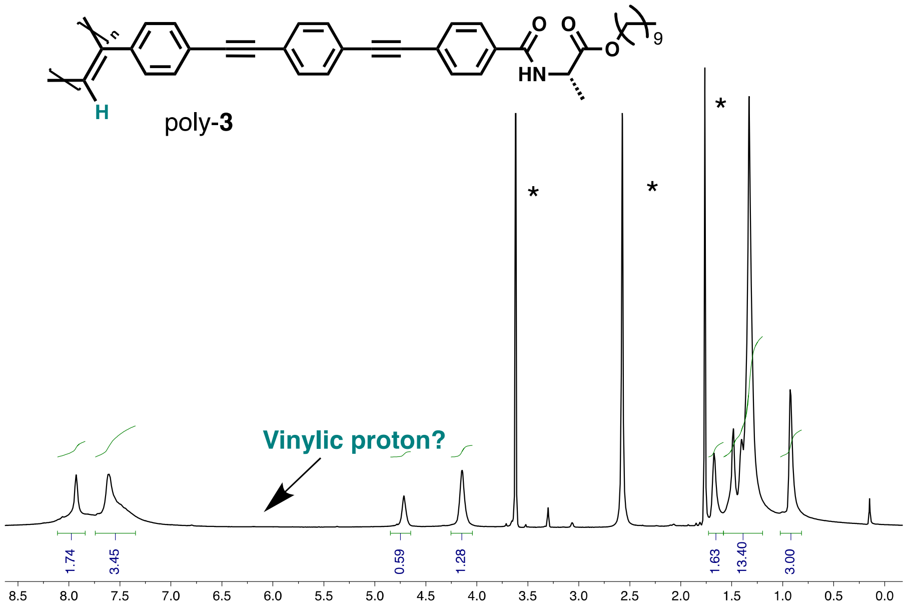

Figure S13. ^{1}H NMR spectra for poly-**3** in THF-d_8.

3.2.2 T_2 Experiments

To demonstrate the difference in mobility of the protons along the helical structure, ^{1}H-NMR experiments were recorded at different transverse relaxation (T_2) times. These studies show a clear dependence of the ^{1}H-NMR signals with the T_2 employed. Hence, the decay for the vinyl protons and the aromatic ring directly attached to the polyene backbone is too fast compared to that of the protons of the chiral pendant group. The rapid decay of the NMR signal for the vynil and the aromatic protons of the first aryl ring makes that the signals appear very broaden or remain hidden (NMR invisible or silent). This explains why the integrated area does not match the expected one and makes not possible to calculate the % *cis* content by using ^{1}H-NMR experiments.

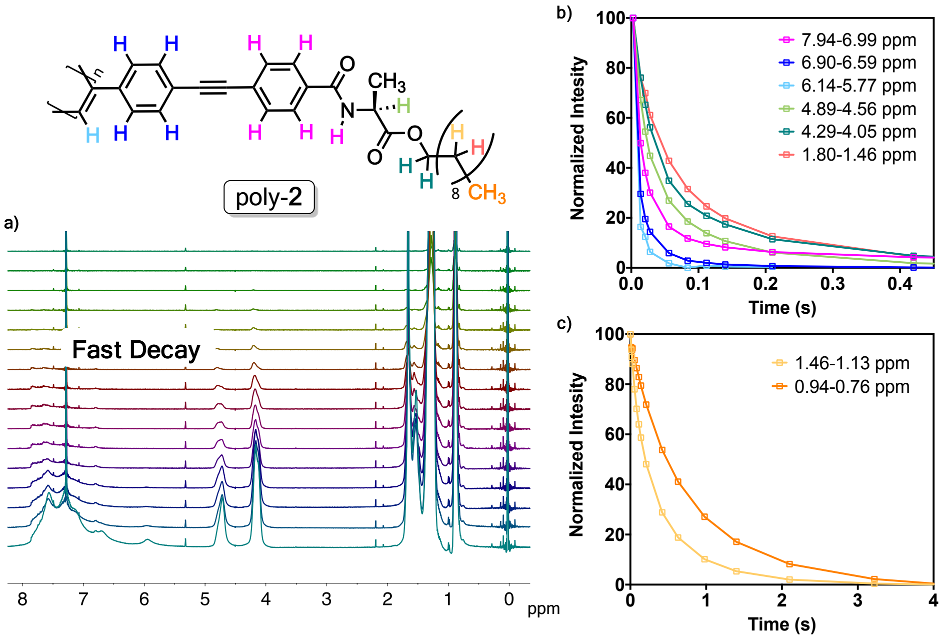

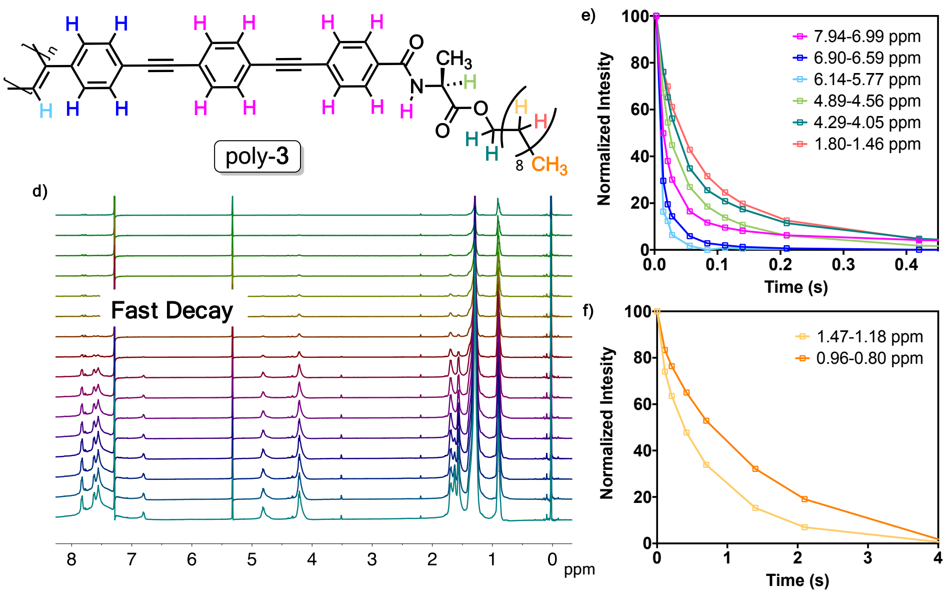

Figure S14. ^{1}H T_2 relaxation spectra for (a) poly-**2** and (d) poly-**3** recorded with increasing relaxation times (from bottom to top). T_2 relaxation decay representation for each proton of (b,c) poly-**2** and (d,e) poly-**3**.

3.2.3 STD NMR Experiments

In order to corroborate that fractions of NMR signals remain hidden, Saturation-Transfer Difference (STD) measurements were carried out at different chemical shifts —5.80 ppm (vinylic band) and 7.00 ppm (aryl ring closer to the polyene backbone)—. By using this technique, a proton signal can be selectively saturated, followed by an effective spin diffusion through out the protons network directly attached to the irradiated signal. Therefore, if there are hidden protons in the baseline, the magnetization is propagated across the entire network of protons in large macromolecules and will become visible. On the other hand, if no protons are hidden at the irradiated frequencies, no signal is observed as the saturation cannot be difussed.[S2]

For poly-**2** saturation at 7.00 ppm reveals that the aryl group closer to the polyene backbone is connected to the vinyl proton and the signals adscribed to the pendant moiety show a reduce in intensity, as the protons are placed far away for an effective spin difussion. The proximity between the vinyl proton and the phenyl group is confirmed by irradiating at 5.80 ppm, observing that the more intense peaks are for the benzene groups.

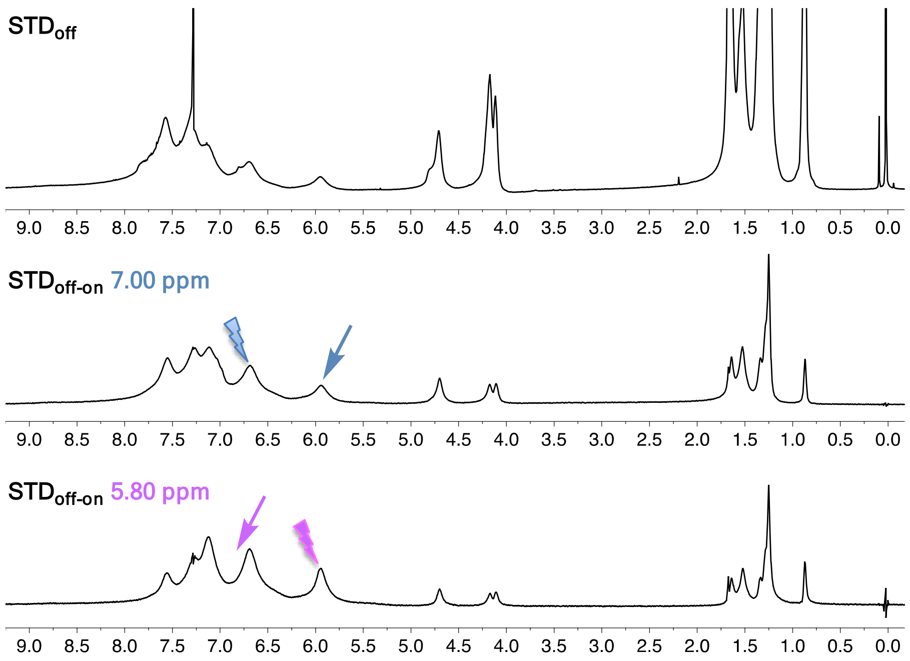

Figure S15. Comparision of the ^{1}H NMR STD spectra for poly-**2** in $CDCl_3$ before and after irradiation at 7.00 ppm and 5.80 ppm (from top to bottom).

In an analogous way, STD ^{1}H NMR experiments were carried out for poly-**2** in different $CDCl_3/CCl_4$ mixture ratios and in pure CCl_4. In all cases the recorded spectra confirmed the proximity between the vinyl proton and the aryl ring.

a)

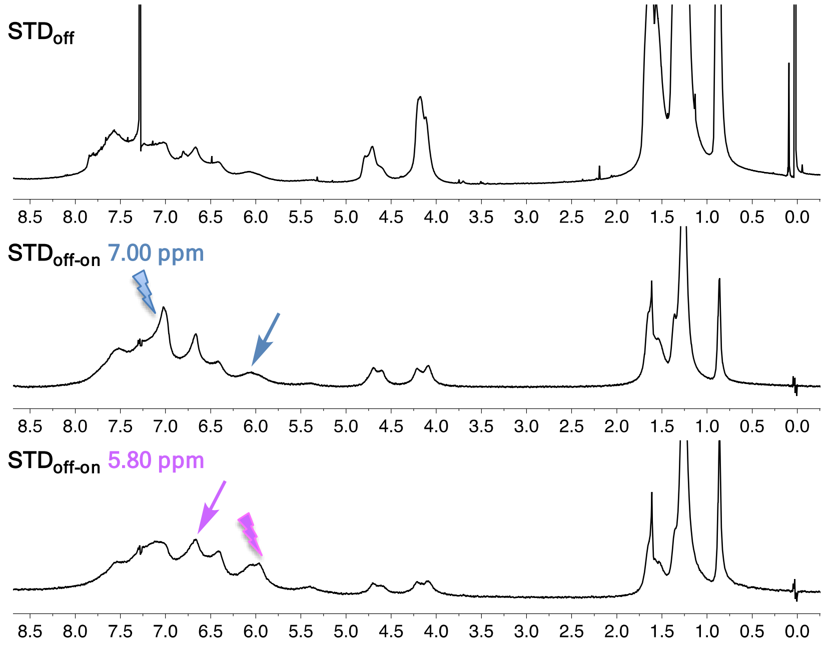

b)

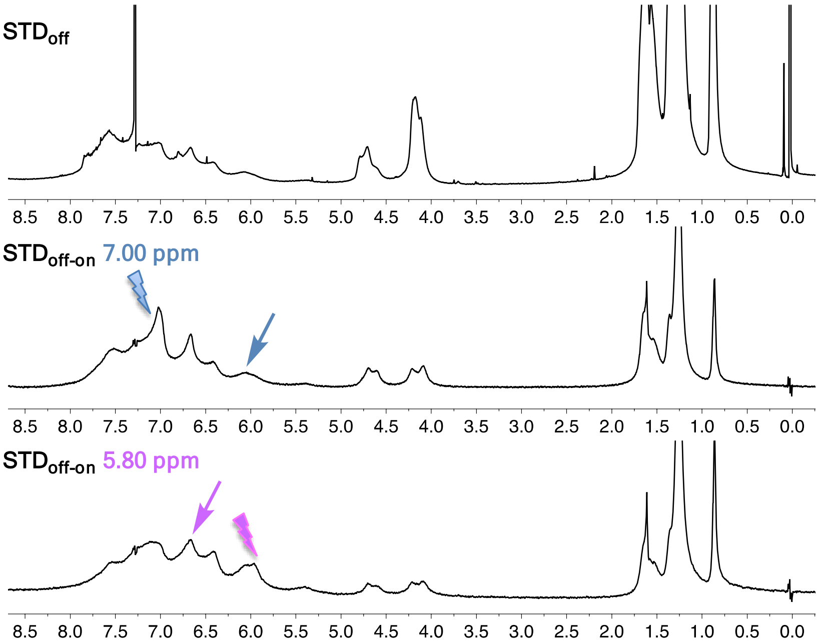
STDoff
8.5 8.0 7.5 7.0 6.5 6.0 5.5 5.0 4.5 4.0 3.5 3.0 2.5 2.0 1.5 1.0 0.5 0.0
STDoff-on 7.00 ppm
8.5 8.0 7.5 7.0 6.5 6.0 5.5 5.0 4.5 4.0 3.5 3.0 2.5 2.0 1.5 1.0 0.5 0.0
STDoff-on 5.80 ppm
8.5 8.0 7.5 7.0 6.5 6.0 5.5 5.0 4.5 4.0 3.5 3.0 2.5 2.0 1.5 1.0 0.5 0.0

c)

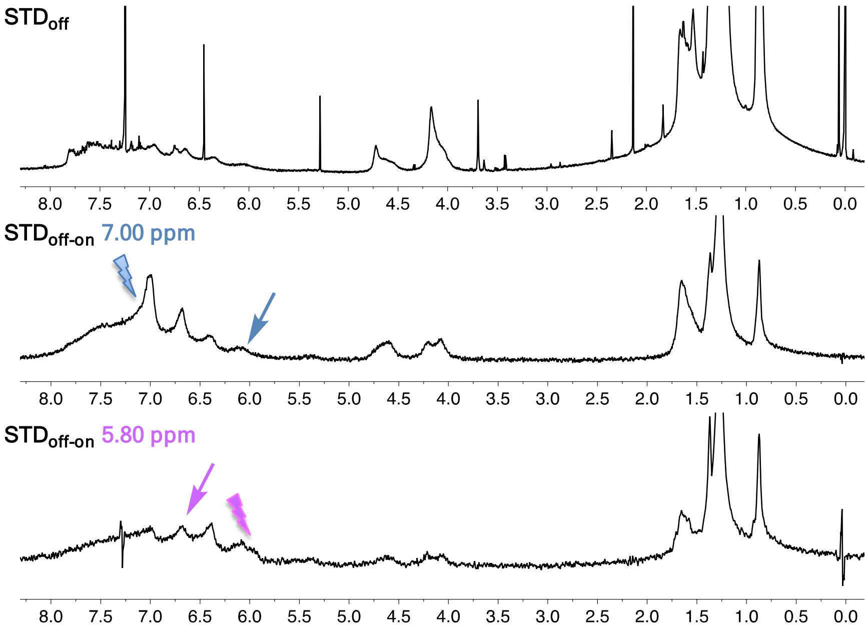
STDoff
8.0 7.5 7.0 6.5 6.0 5.5 5.0 4.5 4.0 3.5 3.0 2.5 2.0 1.5 1.0 0.5 0.0
STDoff-on 7.00 ppm
8.0 7.5 7.0 6.5 6.0 5.5 5.0 4.5 4.0 3.5 3.0 2.5 2.0 1.5 1.0 0.5 0.0
STDoff-on 5.80 ppm
8.0 7.5 7.0 6.5 6.0 5.5 5.0 4.5 4.0 3.5 3.0 2.5 2.0 1.5 1.0 0.5 0.0

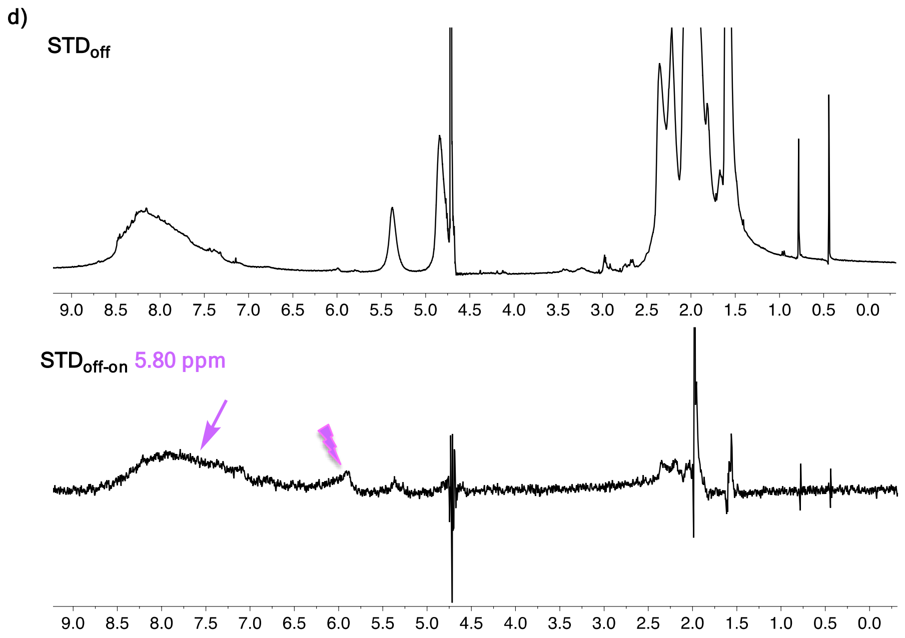

Figure S16. Comparision of the ^{1}H NMR STD spectra for poly-**2** in (a) 75% $CDCl_3$:25% CCl_4, (b) 50% $CDCl_3$:50% CCl_4 and (c) 25% $CDCl_3$:75% CCl_4 before and after irradiation at 7.00 ppm and 5.80 ppm (from top to bottom). (d) ^{1}H NMR STD spectra for poly-**2** in CCl_4 before and after irradiation at 5.80 ppm.

Similar to poly-**2**, poly-**3** was saturated at two different NMR shifts. By selectively irradiating the signal at 7.40 ppm, a signal not previously observed at the ^{1}H NMR STD_{off} appears at 6.15 ppm, which can be ascribed to the vinylic proton. STD experiments at 6.10 ppm reveal that this signal is directly related to the benzene rings, indicating that the proton observed at 6.15 ppm corresponds to the vinylic one. This peak is not observed due to the broadening of the signal, which makes it remain hidden in the baseline.

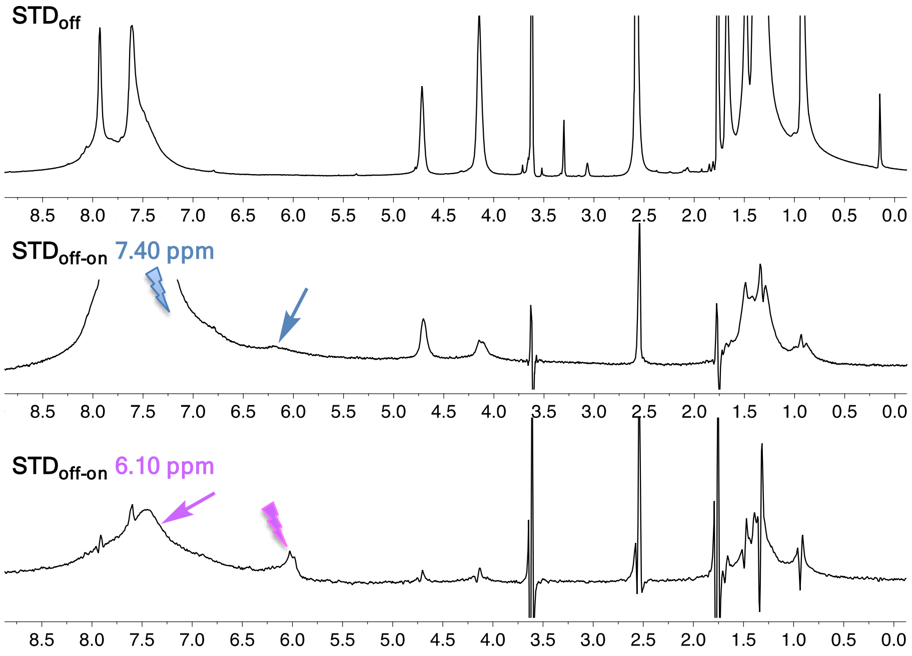

Figure S17. Comparision of ^{1}H NMR STD experiments for poly-**3** in $CDCl_3$ before and after irradiation at 7.40 ppm and 6.10 ppm (from top to bottom).

3.3 Raman Experiments

The bands observed by Raman resonance confirmed the *cis* configuration. The peak at highest wavelength corresponds to the C=C bond stretching and overlaps with that of the phenyl ring. The band at 1350-1340 cm^{-1} arises from the *cis* C-C bond coupled with the single bond connecting the main chain and the phenyl ring. The peak at lowest wavelength corresponds to the C-H bond of the *cis* form. The disappearance of the alkyne band (ca. 2110 cm^{-1}) confirms the formation of the conjugated double bonds of the polymer backbone.

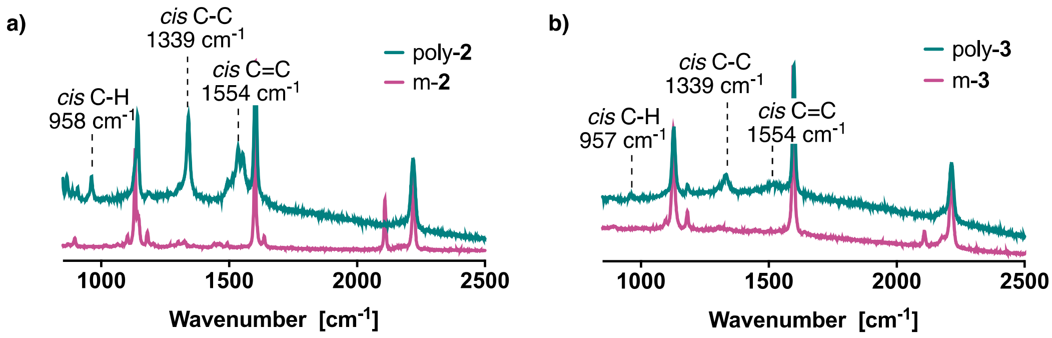

Figure S18. Overlay of the Raman spectra obtained for (a) m-**2** and poly-**2** and (b) m-**3** and poly-**3**.

4. GPC Studies

The molecular weight was estimated by GPC using THF (flow rate: 1.0 mL min^{-1}) as eluent and polystyrene narrow standards (PSS) as calibrants.

Table S2. GPC data for the synthesized polymers.

Polymer	Mn	Mw	Mz	PDI	DP
poly-**2**	10143	13015	16113	1.28	28
poly-**3**	8827	10899	13275	1.23	19

4.1 Polymers Stability Studied by GPC

To corroborate the stability of poly-**2** and poly-**3**, GPC experiments were mesured before and after one week. The obtained traces are almost coincident to those initially obtained, indicating that these polymers are stable in solution.

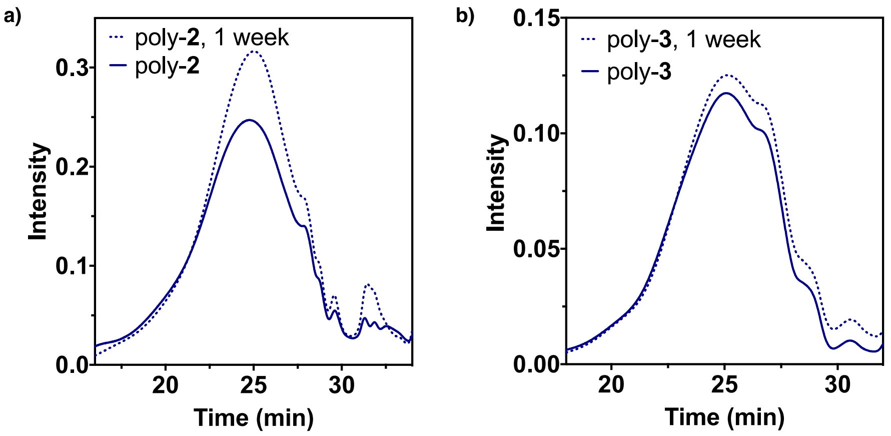

Figure S19. GPC traces comparison for (a) poly-**2** and (b) poly-**3** freshly prepared samples and the same samples after one week.

5. TGA Studies

The thermal stability was evaluated by TGA. The degradation starts at 300 ºC for poly-**2** and for poly-**3** at 310 ºC.

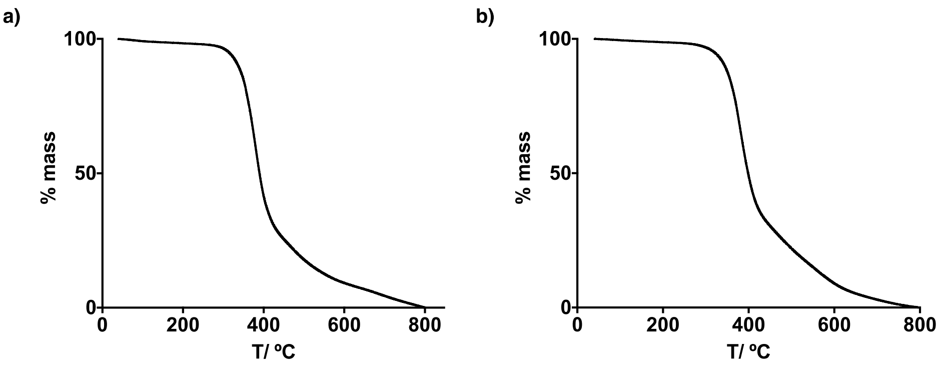

Figure S20. TGA traces for (a) poly-**2**, (b) poly-**3**.

6. Dynamic Beahviour

6.1 Studies for poly-2

6.1.1 Analysis in Different Solvents

The CD spectra for poly-**2** were recorded in different solvents at a 0.44 mM concentration. The different scaffolds adopted —classical *c-t* helix, ω_1=165º, and matryoshka-like helix, ω_1= 170º— can be easily tracked by UV-Vis as well as by CD spectroscopy.

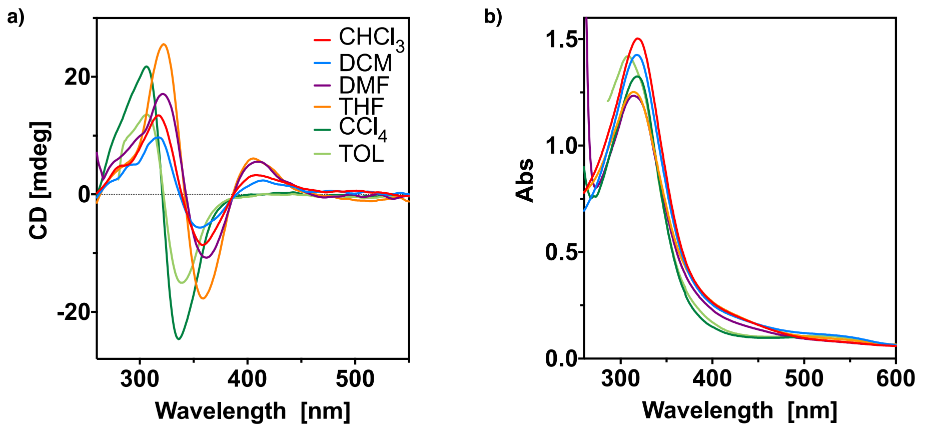

Figure S21. (a) CD and (b) UV-Vis spectra for poly-**2** (0.44 mM, 25 ºC) in different solvents.

6.1.2 Raman Experiments for the Elongated and Stretched Scaffolds

The stretching observed by UV-Vis for low-polar solvents is confirmed by Raman spectroscopy. If poly-**2** is dissolved in $CHCl_3$ (stretched polymer) and in CCl_4 (elongated polymer), a shift of 7 nm in the vibrational band associated to the cis C-H stretching (ca., 1000 nm^{-1}) is observed.

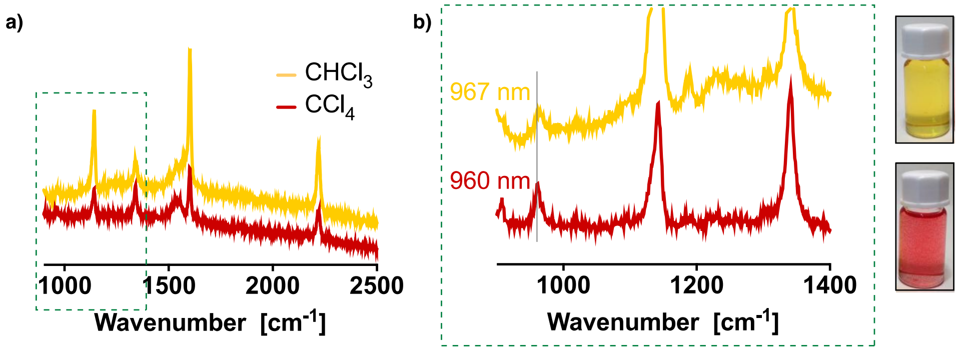

Figure S22. (a) Raman spectra obtained when poly-**2** is dissolved in $CHCl_3$ and CCl_4. (b) Magnification of the 900 to 1400 cm^{-1} area highlighted in (a) and vials depicting visual changes in the colour solutions caused by the different helical scaffolds adopted by poly-**2**.

6.1.3 Interaction with Metal Ions

The addition of metal ions produces no chiral enhancement or helical inversion, which indicates that, although the metal ions can attach to the chiral moiety, no effect is produced in the helical scaffold. This is probably due to the supramolecular interactions established between pendants that forbid any changes in the polymer.

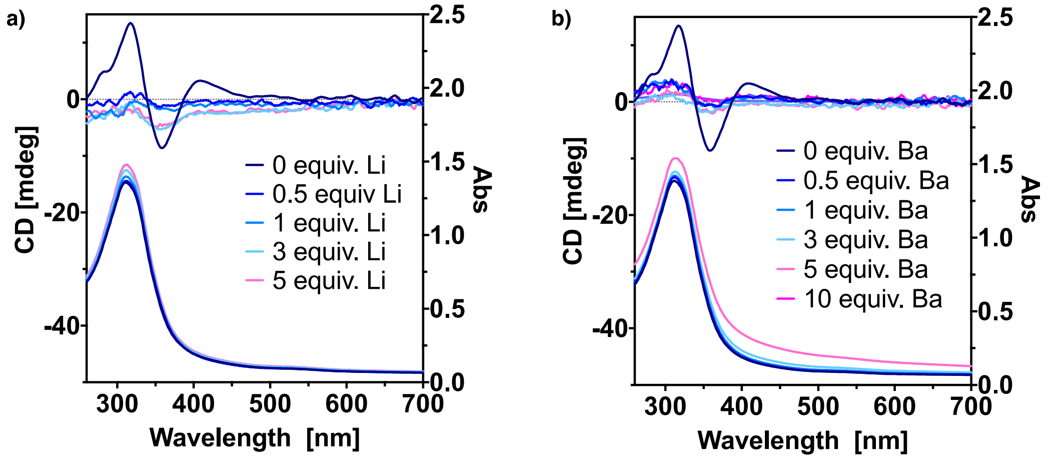

Figure S23. ECD and UV-Vis spectra for poly-**2** (0.44 mM, $CHCl_3$, 25 ºC) after the addition of (a) $LiClO_4$ (470 mM, THF) and (b) $Ba(ClO_4)_2$ salts (149 mM, THF).

6.2 Studies for poly-3

6.2.1 Analysis in Different Solvents

To prepare the polymer samples, due to the difficulty of solubilizing the polymer, small ammounts of poly-**3** were weighted and dissolved in the corresponding solvent at the desired concentration (0.36 mM). The resulting mixture was heated overnight at 40 ºC while being magnetically stirred.

Depending on the dielectric constant of the solvent in which poly-**3** is dissolved, it will adopt an *M* or *P* helix. The change in the conformation of the chiral moiety is produced when $(\varepsilon-1)/(\varepsilon+1) > 0.8$.

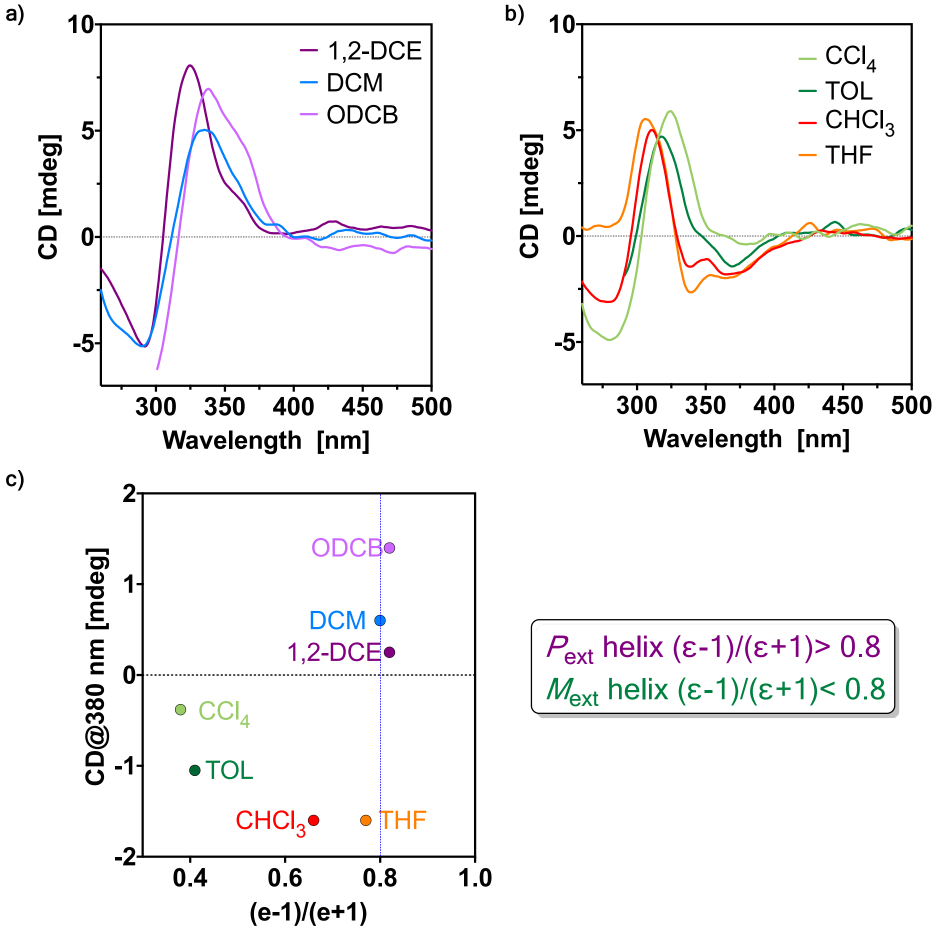

Figure S24. (a) and (b) CD spectra for poly-**3** (0.36 mM, 25 ºC) evaluated in different solvents. (c) Plot of the CD intensity of poly-**3** at 380 nm as a function of the dielectric constant of the different solvents.

6.2.2 Interaction with Metal Ions and Effect of the Temperature

For poly-**3** a chiral inversion is produced after the addition of $LiClO_4$ salts, while the addition of Ba^{2+} produced no changes. VT-CD experiments highlight the role played by the strong non-covalent interactions established among pendants, as no changes are produced when heating a solution of poly-**3** in DCM (Figure S23c) or THF (Figure S23d).

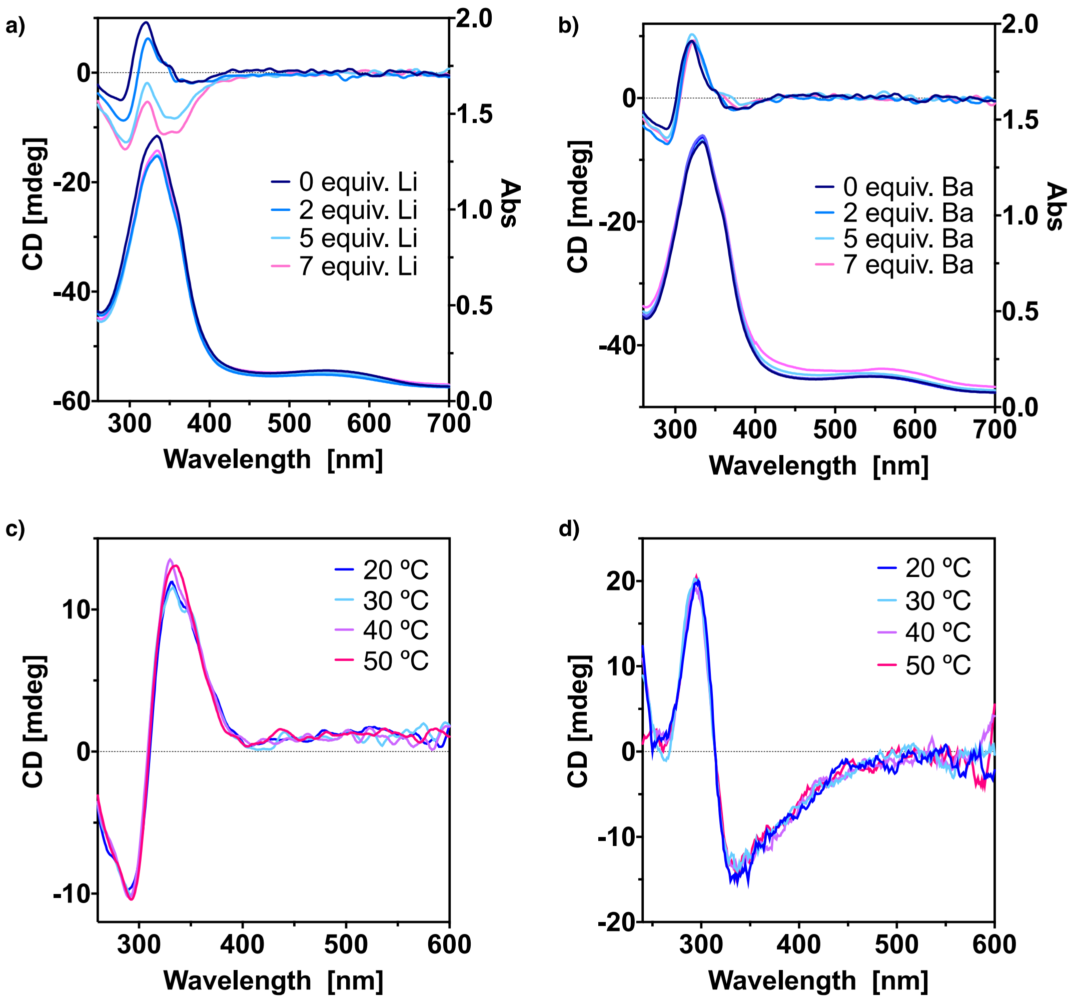

Figure S25. Metal ion titration for poly-3 (0.72 mM, CHCl3, 25 ºC) with (a) LiClO4 (470 mM, THF) and (b) Ba(ClO4)2 (140 mM, THF). VT-CD studies for poly-3 in (c) DCM (0.72 mM) and (d) THF (0.72 mM).

7. Formation of SP-3

Polymer solutions of SP-**3** were prepared by weighting the monomer and adding the necessary ammount of MCH to obtain the desired concentration (0.15 mM). To completetly solubilize the monomer the mixture was heated up to 80 ºC for two hours.

A similar protocol was used to obtain m-**3**. In this case, the monomer presents no solubility problems in medium polar or polar solvents, such as DCM, and, as a consequence, the sample does not need to be heated.

The large solubility of m-**3** in polar solvents prevents the formation of the aggregate and this can be easily observed by ECD spectroscopy. If the monomer is dissolved in DCM a null ECD trace is obtained, whereas if it is dissolved in MCH an active ECD is produced, indicative of the formation of a chiral aggregate (Figure 24a). The formation of the aggregate (SP-**3**) is also confirmed by UV-Vis spectroscopy (Figure 24b).

Contrary to poly-**3**, poly-**2** produced no supramolecular aggregate.

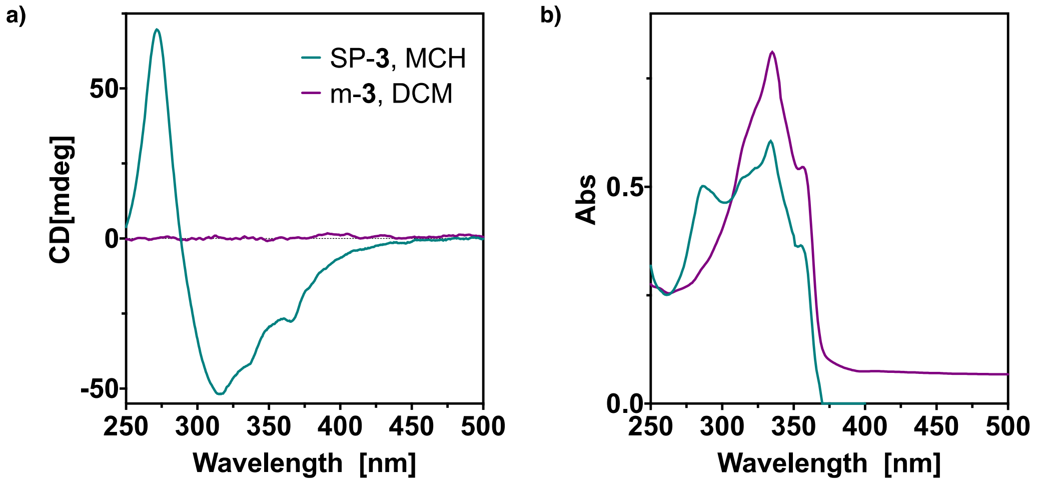

Figure S26. (a) CD and (b) UV-Vis comparison for SP-**3** and m-**3** dissolved in DCM (0.15 mM, 25 ºC).

8. Atomic Force Microscopy (AFM) Measurements for poly-2

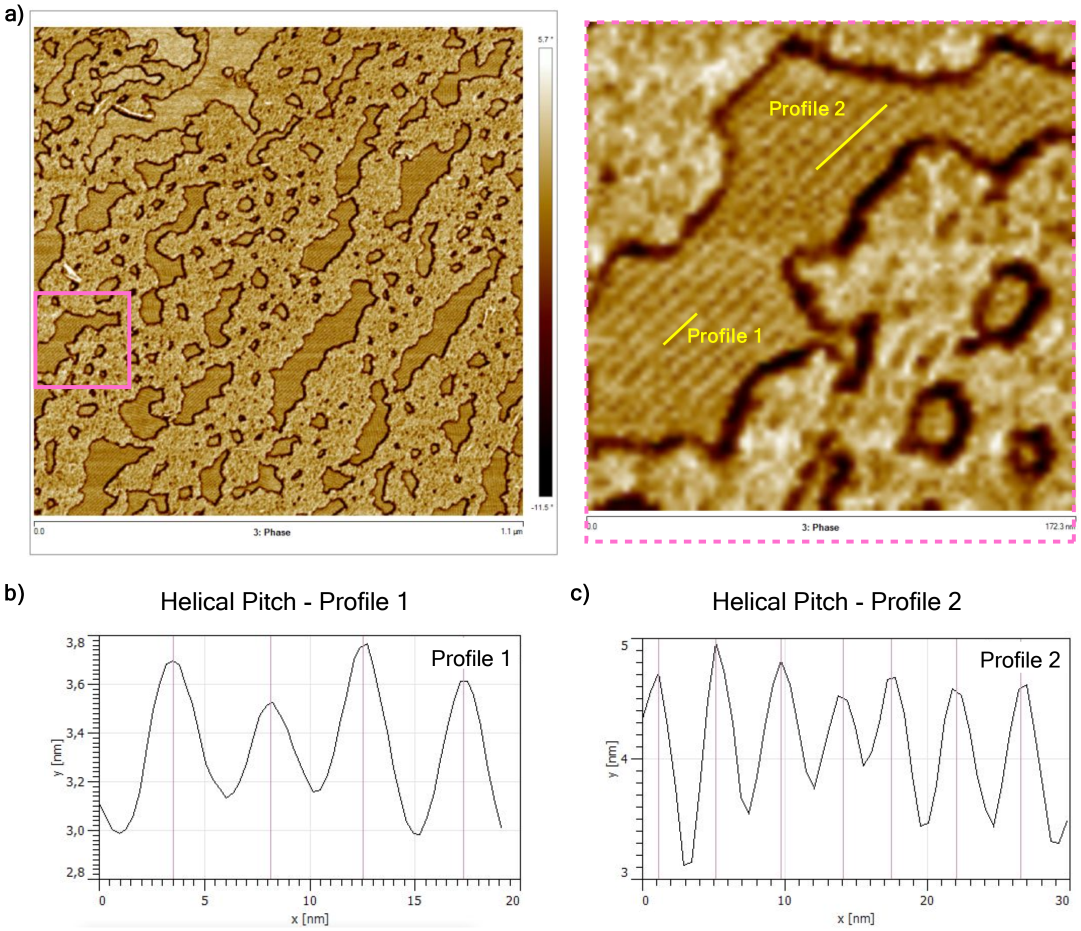

Points	X [nm]	Y [pm]	Length [nm]
	3,52	3698	
	8,14	3525	4,63
	12,53	3762	4,39
	17,30	3616	4,77

Points	X [nm]	Y [nm]	Length [nm]
	1,13	4,721	
	5,14	4,961	4,01
	9,72	4,818	4,58
	14,08	4,509	4,37
	17,46	4,674	3,38
	22,04	4,566	4,58
	26,55	4,594	4,51

Figure S27. (a) AFM image for poly-**2** and zoomed area or the highlighted region. (b,c) Graphics depicting the helical pitch profile measured in the indicated areas.

9. SAXS Experiments

The obtained 1D SAXS profile for poly-**2** shows a single peak at around 4.0 nm. This value is in close to that obtained by AFM for the helical pitch, confirming that the 3D model propossed is a good approximation.

In the case of poly-**3**, four peaks can be observed. The smallest values —2.859 and 1.858 nm— correspond to the non covalent interactions established between pendants, mainly hydrogen bonding and π-π stacking. The largest values —5.560 and 12.928 nm— are ascribed to the helix width and the helical pitch, respectively. This data is coincident with the one obatined for the 3D model of poly-**3**.

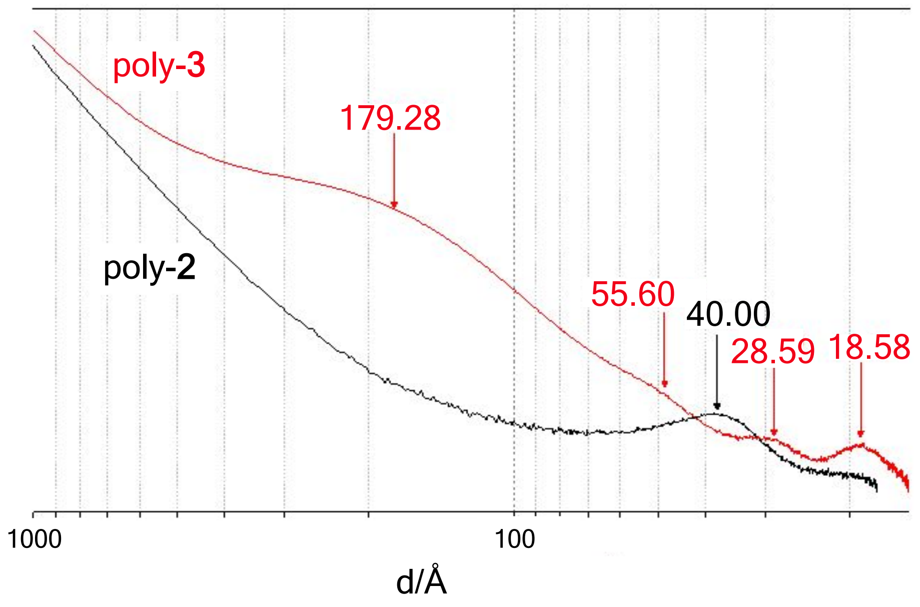

Figure S28. 1D SAXS profile for poly-**2** and poly-**3**.

10. Theoretical Calculations

Considering the difficulties to carry out ECD theoretical calculations on large polymers, representative oligomers were used.

On the one hand, in the case of poly-**2** an oligomer with n= 8 —where n denotes the number of monomer repeating units (mru)— was employed and, in order to reduce the computational demands, the long alkyl chains were replaced by methyl groups. The number of monomer units was selected considering the results of previous studies,[S3-S5] where we evaluated the spectra for a series of poly(phenylacetylene) (PPA) oligomers obtained through systematic increase of monomer units, and concluded that 8-10 monomers were enough to describe the n+2 polymer ECD spectra. The starting structure of poly-**2** was built through adjustment to the experimental data obtained from structural techniques, such as AFM and UV-Vis spectroscopy, defining the four different dihedral angles needed to build up the helical scaffold (ω_1, ω_2, ω_3 and ω_4; see Figure S25). Additionally, the pendant groups were introduced in the most stable conformation (*ap*). The oligomer geometry was optimized using the DFT method[S6] together with the B3LYP-D3 functional[S7] and the 6-31G** basis set.[S8]

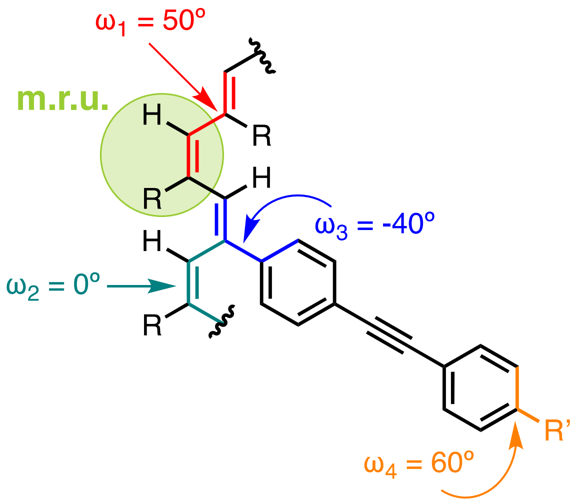

Figure S27. Main dihedral angles involved in the helical structure for PPAs and derivatives.

On the other hand, for poly-**3** the chiral moiety was removed from the pendant units in order to achieve a longer oligomer (n= 20, 504 atoms). In this case the length of the oligomer is essential to visualize the helices described by the OPE pendants (helix 3 and helix 4).

The ECD computational methodology was selected according to the size of polymers under investigation. Taking this into account, to evaluate the theoretical spectra time dependent density functional theory (TD-DFT),[S9] in combination with the CAM-B3LYP functional[S10] and the 3-21G basis set,[S11] have been used. The ECD calculations were performed with the ORCA program (including 80 excitations).[S12] The Gabedit[S13] code was used to plot the spectra and the

density differences were displayed with Avogadro.[S14] Furthermore, the full width at half height (FWHM) was fixed to 20.0 nm and the ECD were plotted with Gaussian curves.

For an efficient comparison and taking into account the tendency of the TD-DFT method to overestimate the excitation energies, the wavelength and intensity at the maximum/minimum Cotton effect correspondent to the polyene backbone in the theoretical spectra were adjusted to the experimental spectra. Employing the same correction factors, the lambdas were shifted and the intensities rescaled. The resulting ECD spectra are in good agreement with the experimental ones.

Poly-**2** (ω_1 ca. 165º) displays a classical ECD trace with a positive Cotton effect at ca. 405 nm, dominated by the S_0 to S_1 excitation and ascribed to the polyenic backbone (Figure S29).

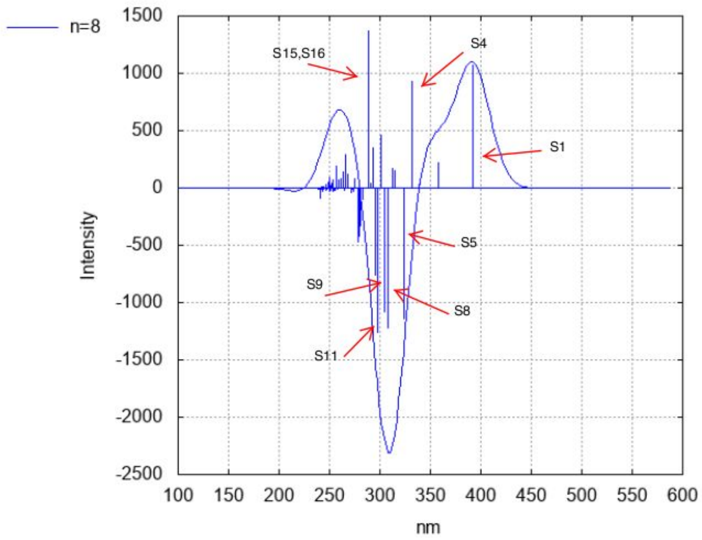

Figure S29. TD-DFT (CAM-B3LYP)/3-21G ECD spectrum for poly-**2** evaluated at the DFT(B3LYP-D3)/6-31G** geometries showing the excited states that contribute the most to the Cotton bands.

To get more insight into these spectral bands, the electron density differences for the corresponding transitions were evaluated at the same level of theory (Figure S30).

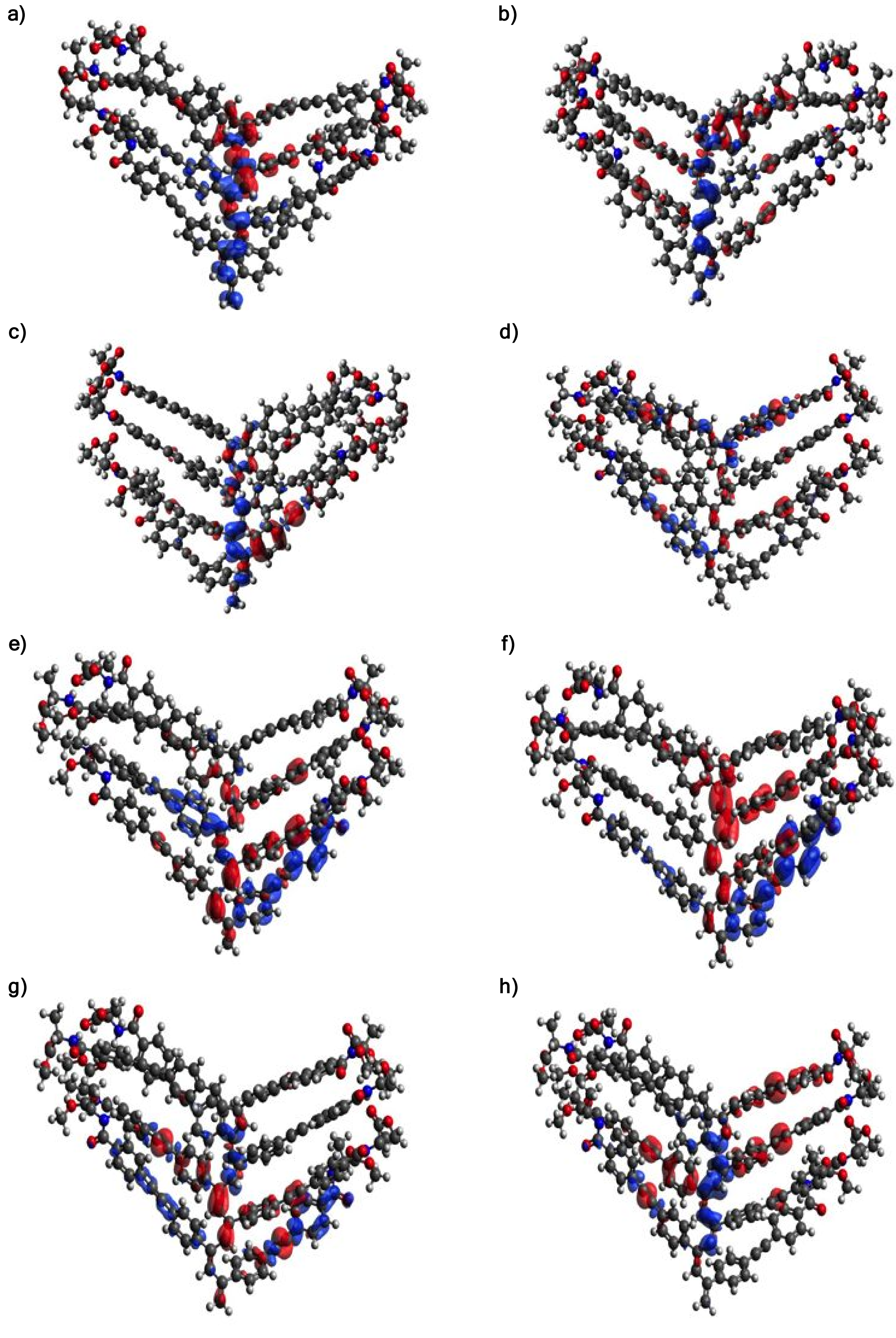

Figure S30. Electron density differences with respect to the ground state for (a) S0 to S1, (b) S0 to S4, (c) S0 to S5, (d) S0 to S8, (e) S0 to S9, (f) S0 to S11, (g) S0 to S15 and (h) S0 to S16. An isovalue of 0.0005 was selected.

Poly-**3** (ω_1 ca. 170º) ECD shows a highly intense positive Cotton effect at ca. 340 nm, dominated by the S_0 to S_1 excitation and ascribed to the polyenic backbone. The excited states that mostly contribute to the Cotton bands are depicted in Figure S31.

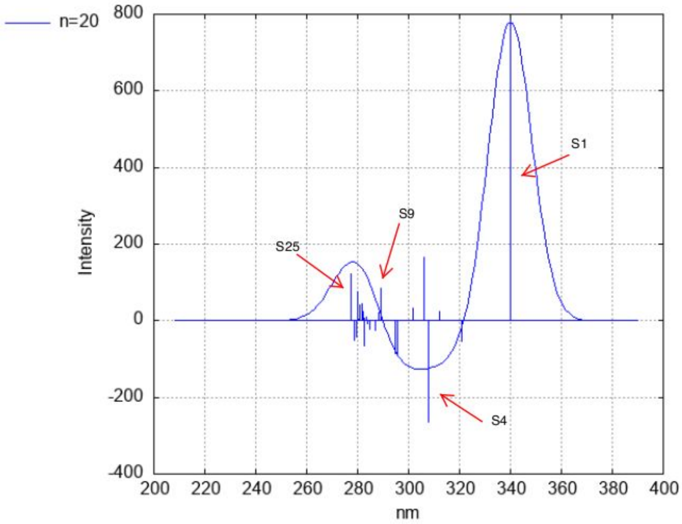

Figure S31. TD-DFT (CAM-B3LYP)/3-21G ECD spectrum for poly-**3** evaluated at the DFT(B3LYP-D3)/6-31G** geometries showing the excited states that contribute the most to the Cotton bands.

To gain insight into the spectrum, the corresponding electron density differences were evaluated at the same level of theory (Figure S32).

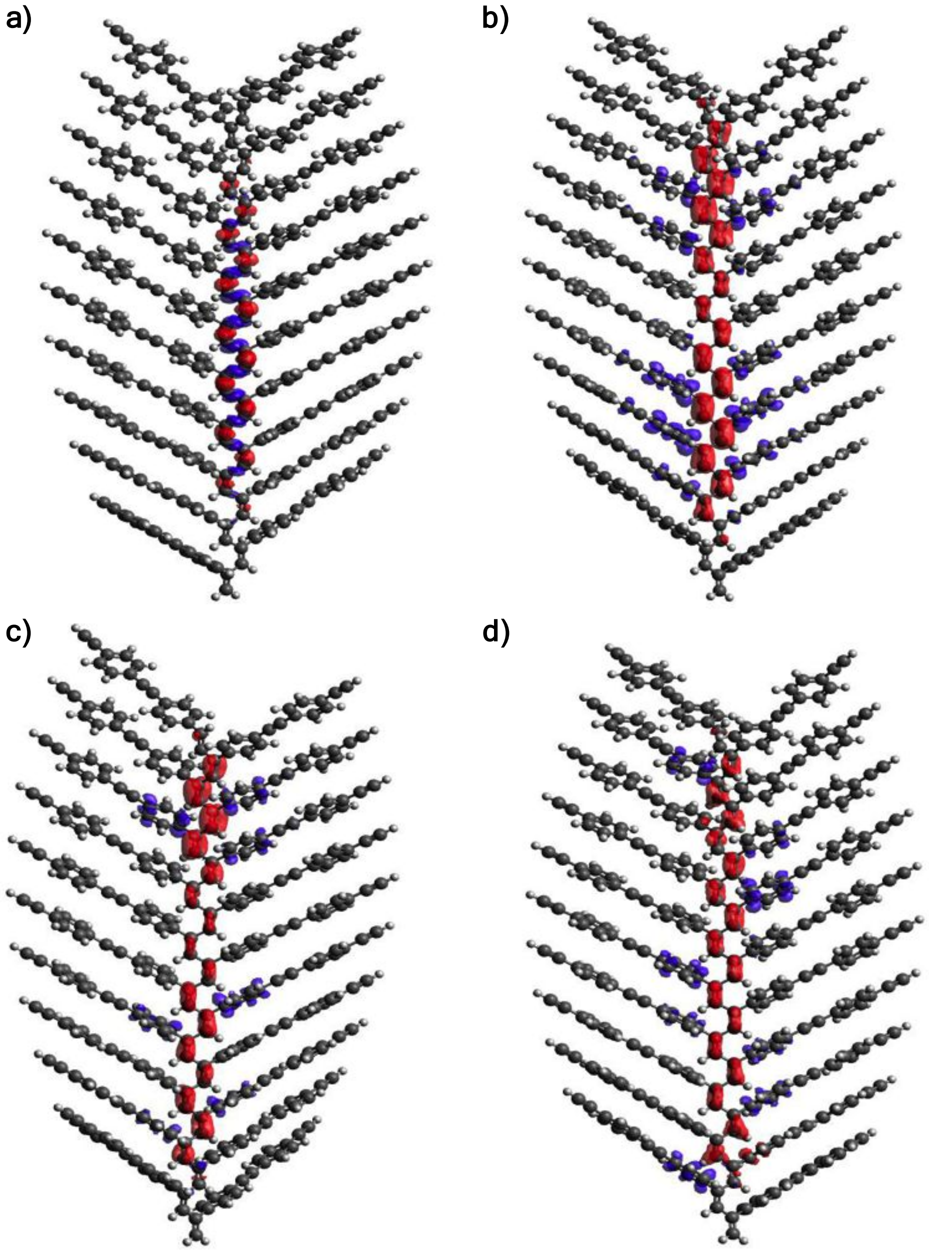

Figure S32. Electron density differences with respect to the ground state for (a) S0 to S1, (b) S0 to S4, (c) S0 to S9, and (d) S0 to S25. An isovalue of 0.0005 was selected.

10. Supporting References

S1. Okoshi, K.; Sakajiri, K.; Kumaki, J.; Yashima, E. Well-Defined Lyotropic Liquid Crystalline Properties of Rigid-Rod Helical Polyacetylenes. *Macromolecules* **2005**, *38*, 4061-4064.

S2. Novoa-Carballal, R.; Martín-Pastor, M.; Fernández-Megía, E. Unveiling an NMR-Invisible Fraction of Polymers in Solution by Saturation Transfer Difference**.** *ACS Macro. Lett.* **2021**, *10*, 1474-1479.

S3. Fernández, B.; Rodríguez, R.; Rizzo, A.; Quiñoá, E.; Riguera, R.; Freire, F. Predicting the Helical Sense of Poly(phenylacetylene)s from their Electron Circular Dichroism Spectra. *Angew. Chem. Int. Ed*. **2018**, *57*, 3666-3670.

S4. Fernández, B.; Rodríguez, R.; Quiñoá, E.; Riguera, R.; Freire, F. Decoding the ECD Spectra of Poly(phenylacetylene)s: Structural Significance*. ACS Omega* **2019**, *4*, 5233-5240.

S5. Fernández, Z.; Fernández, B.; Quiñoá, E.; Riguera, R.; Freire, F. Chiral Information Harvesting in Helical Poly(acetylene) Derivatives using Oligo(*p*-phenyleneethynylene)s as Spacers. *Chem. Commun.* **2020**, *11*, 7182-7187.

S6. Hohenberg, P.; Walter, K. Inhomogeneous Electron Gas. *Physical Review* **1964**, *136,* B864-B871.

S7. Grimme, S. Density Functional Theory with Dispersion Corrections. WIREs Computational Molecular Science **2011**, *1*, 211-228.

S8. (a) Hariharan, P. C.; Pople, J. A. The Influence of Polarization Functions on Molecular Orbital Hydrogenation Energies. Theor. Chim. Acta **1973**, *28*, 213-222. (b) Hehre, W. J.; Ditchfield, R.; Pople, J. A. Self-Consistent Molecular Orbital Methods. XII. Further Extensions of Gaussian-Type Basis Sets for Use in Molecular Orbital Studies of Organic Molecules. *J. Chem. Phys.* **1972**, *56*, 2257-2261.

S9. Runge, E.; Gross, E. K. U. Density-Functional Theory for Time-Dependent Systems. *Phys. Rev. Lett*. **1984**, *52*, 997-1000.

S10. Yanai, Y.; Tew, D. P.; Handy, N. C. A New Hybrid Exchange-Correlation Fuctional Using the Coulomb-attenuating Method (CAM-B3LYP). *Chem. Phys. Lett.* 2005, 393, 51-57.

S11. Binkley, J. S.; Pople, J. A.; Hehre, W. J. Self-consistent Molecular Orbital Methods. 21. Small Split-valence Basis Sets for First-row Elements. *J. Am. Chem. Soc.* **1980**, 102, 939-947.

S12. Neese, F. The ORCA program system. *WIREs Comput. Mol. Sci*. **2012**, *2*, 73-78.

S13. Allouche, A. R. Gabedit - A Graphical User Interface for Computational Chemistry Softwares. *J. Comput. Chem.* **2011**, *32*, 174-182.

S14. Hanwell, M. D.; Curtis, D. E.; Lonie, D. C.; Vandermeersch, T.; Zurek, E.; Hutchison, G. R. Avogadro: an Advanced Semantic Chemical Editor, Visualization, and Analysis Platform. *J. Cheminformatics* **2012**, *4*,17.
